# A spatiotemporally explicit paleoenvironmental framework for the Middle Stone Age of eastern Africa

**DOI:** 10.1038/s41598-022-07742-y

**Published:** 2022-03-07

**Authors:** Lucy Timbrell, Matt Grove, Andrea Manica, Stephen Rucina, James Blinkhorn

**Affiliations:** 1 Department of Archaeology, Classics and Egyptology, University of Liverpool, Liverpool, UK; 2 Department of Zoology, University of Cambridge, Cambridge, UK; 3 Department of Earth Sciences, National Museums of Kenya, Nairobi, Kenya; 4 Pan-African Evolution Research Group, Max Planck Institute for the Science of Human History, Jena, Germany; 5 Department of Geography, Centre for Quaternary Research, Royal Holloway, University of London, Egham, UK

**Keywords:** Archaeology, Cultural evolution, Ecological modelling, Palaeoecology

## Abstract

Eastern Africa has played a prominent role in debates about human evolution and dispersal due to the presence of rich archaeological, palaeoanthropological and palaeoenvironmental records. However, substantial disconnects occur between the spatial and temporal resolutions of these data that complicate their integration. Here, we apply high-resolution climatic simulations of two key parameters, mean annual temperature and precipitation, and a biome model, to produce a highly refined characterisation of the environments inhabited during the eastern African Middle Stone Age. Occupations are typically found in sub-humid climates and landscapes dominated by or including tropical xerophytic shrubland. Marked expansions from these core landscapes include movement into hotter, low-altitude landscapes in Marine Isotope Stage 5 and cooler, high-altitude landscapes in Marine Isotope Stage 3, with the recurrent inhabitation of ecotones between open and forested habitats. Through our use of high-resolution climate models, we demonstrate a significant independent relationship between past precipitation and patterns of Middle Stone Age stone tool production modes overlooked by previous studies. Engagement with these models not only enables spatiotemporally explicit examination of climatic variability across Middle Stone Age occupations in eastern Africa but enables clearer characterisation of the habitats early human populations were adapted to, and how they changed through time.

## Introduction

Fossil, archaeological, and environmental records, together with the geographic placement of the region, suggest that eastern Africa played a prominent role in the evolution and dispersal of our species, *Homo sapiens*^1^. The mosaic morphology of recently discovered Mtoto fossils at Panga Ya Saidi demonstrates that eastern Africa sustained great diversity amongst Middle Stone Age (MSA) populations into the Late Pleistocene^2^, supporting a pan-African origin for *H. sapiens* rather than a single centre of endemism^3,4^. Across the continent, the MSA is becoming increasingly associated with the emergence of our species due to the near-simultaneous appearance of cultural innovations diagnostic of the MSA and hominin fossils possessing ‘modern’ features autapomorphic to *H. sapiens*^5–8^*.* The MSA archaeology record in eastern Africa is particularly rich compared to other regions, largely because of an extensive research history combining extensive use of chronometric dating and controlled excavation as well as its ideal conditions for preservation. This makes it an essential testing ground for hypotheses regarding modern human behavioural evolution^9–13^, especially in relation to palaeoclimatic change^14–17^.

**Figure 1:**
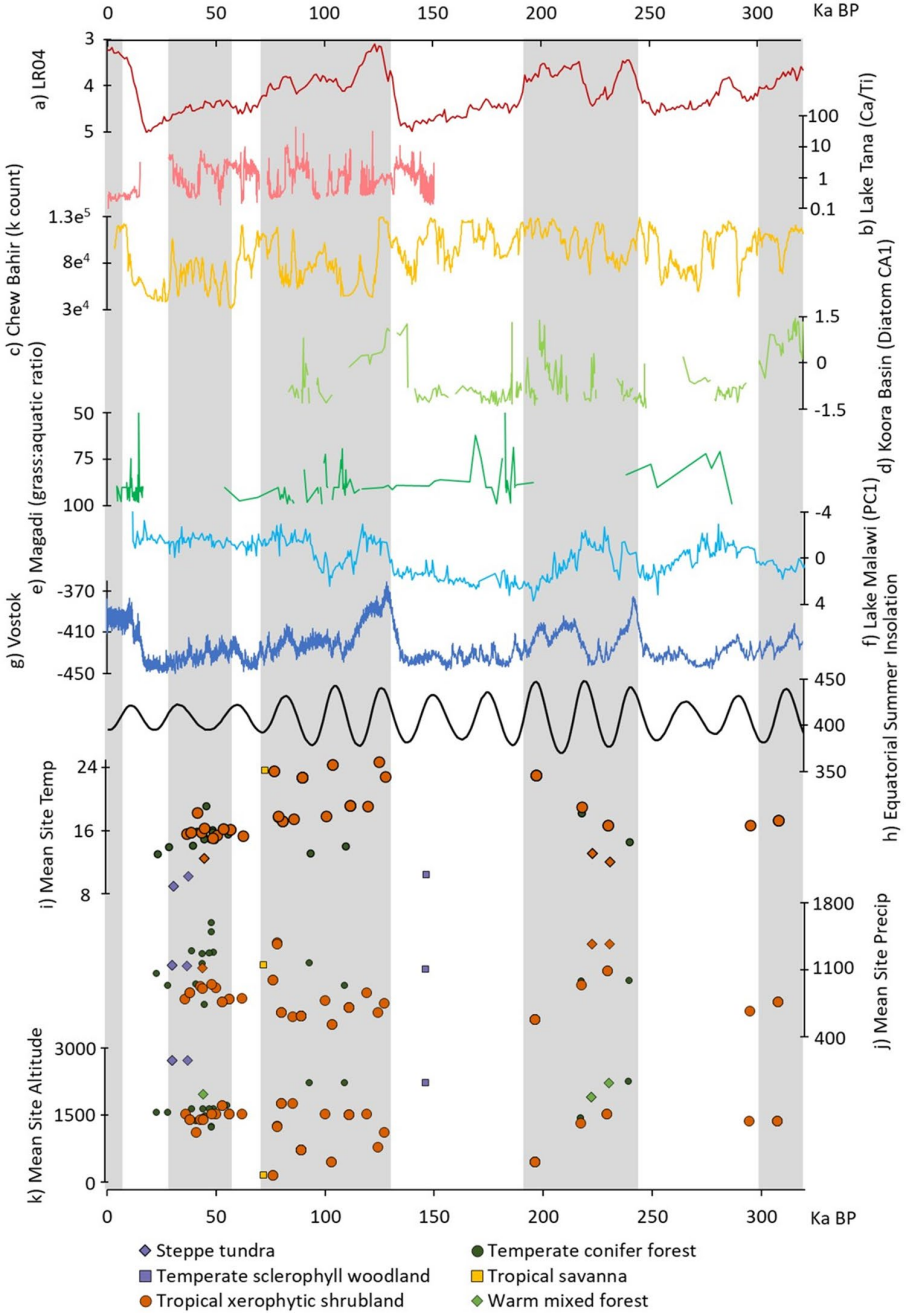
Composite of key inter-regional and regional climatic records, insolation, and temperature, precipitation and altitude parameters for dated eastern African Middle Stone Age occupations including (**a**) LR04 Benthic Stack δ^18^O^25^; (**b**) Lake Tana Ca/Ti ratio^19^; (**c**) Chew Bahir k-counts^26^; (**d**) Koora Basin diatom CA^20^; (**e**) Magadi Pollen grass:aquatic ratio^27^; (**f**) Lake Malawi PC1^28^; (**g**) Vostok δD [‰ SMOW]^29^; (**h**) mean Equatorial summer (Jun-Aug) insolation (W/m2)^30^; mean (**i**) temperature (°C), (**j**) precipitation (mm), and (**k**) altitude (meters above sea level) within 50 km radius of eastern African MSA assemblages, illustrated at the mid-age point for each occupation and symbolised according to its biome classification. Grey vertical bars indicate odd-numbered Marine Isotope Stages.

Historically, there have been relatively scarce terrestrial climate records from eastern Africa, particularly in contrast to the richness of the archaeological record^18^. Over the past decade, recently assembled lacustrine records now provide evidence for climatic changes in several major basins spanning the region. The near-continuous core record from Lake Tana demonstrates increased climatic fluctuation towards the end of the penultimate glacial period, followed by abrupt change to stable humid conditions during Marine Isotope Stage (MIS) 5e-c^19^. The Lake Tana record, from which Ca/Ti elemental ratios are used as a proxy for effective moisture, has been used to assess Out of Africa dispersal chronologies^15^. However, the lack of stratigraphically secure archaeological records proximate to the core site limits the potential to make strong links between dispersals and climate change. Elsewhere, at Olorgesailie, high-resolution core data is associated with Acheulean and MSA deposits just 24 km away from the drill site, allowing for the identification of a period of increased variability coinciding with the origin of the MSA^20,21^. This is supported by the pollen records at nearby Lake Magadi where the general trend of increased aridification for this period is also reported^22^. Late Pleistocene records from the Chew Bahir basin similarly demonstrate a series of wet-dry transitions of varying amplitude and duration through K counts^23^, with the early modern human fossil site Omo Kibish lying close to (but not within) the basin. This record therefore could potentially offer a climatic context for MSA occupations of the site, considering that highly favourable humid conditions are reported at 200–125,000 years ago (ka)^23^ which broadly corresponds to the new date of the Omo fossils of 212 ± 16 ka^24^. Despite this improvement in resolution, paleoclimatic patterns and the records used to generate them are diverse (demonstrated in Fig. 1) and rooted in a small number of lake basins, complicating their use with the widely distributed MSA records in the region.

Archaeological studies of the eastern African MSA have noted a link between the distribution of sites, behavioural diversity, and environmental conditions^9–13^. Basell^9^ made a key contribution by integrating modelled ecological data with a landmark synthesis of MSA assemblages. This highlighted the placement of MSA site locations near to but not necessarily within wooded ecologies, stressing the importance of ecotonal settings for enduring habitability in the region. Tryon and Faith^13^ made important advances in the quantitative assessment of eastern African MSA lithic assemblage composition, though they lacked the means to make similar developments in the characterisation of MSA environments, acknowledging the mismatched geographies and scales of archaeological and environmental records^18^. Blinkhorn and Grove^10,11^ in a suite of quantitative analyses, brought together ecology and archaeology using an expanded dataset of MSA lithic artefact typology alongside modern environmental datasets and models for the Last Glacial Maximum (LGM) and Last Interglacial (MIS 5e). Their results stressed the influence of environmental settings over the spatiotemporal distribution of MSA sites, with pulsed patterns of occupation intensity occurring during interglacial stages^10^. Significant relationships between raw material and stone tool types and geographic and environmental variables were reported^11^, with the strongest relationships with topographic ‘roughness’, emulating earlier hypotheses by King and Bailey^31^. These results validate the emphasis placed by earlier studies on ecology and the physical landscape^12^; however, it remains to be tested whether Blinkhorn and Grove’s^10,11^ use of arid and humid models as representations of the extremes of variability observed within an interglacial-glacial cycle effectively characterise change throughout the MSA.

As such, our understanding of Middle to Late Pleistocene diachronic change has been greatly improved by high-resolution climatic records, whilst a series of regional syntheses of MSA assemblages has encouraged considerations of the ecological implications of prehistoric climate change^9^, the quantitative assessment of MSA archaeology^13^, and their integration^10,11^. However, as also stressed in a recent review by Patalano et al*.*^32^, lake cores offer very limited insights into spatial patterns of climate change due to the inability to extrapolate across the wider landscape, the difficulty of translating relative wet and dry proxies into tangible climatic or ecological parameters, the non-linear relationship between different proxies and the phenomena they represent and the lack of directly associated, stratigraphically secure archaeological assemblages. Conversely, studies that rely on simplistic arid-humid simulated datasets^9–11^ or use modern biogeographic zones to represent Pleistocene environments^13^ lack chronological precision, and assume uniformity across successive glacial / interglacial cycles. Here we overcome such limitations to provide a sophisticated examination of the distribution and diversity of eastern African MSA occupations through time and space within the context of Middle to Late Pleistocene climate change. We integrate novel high-resolution climate and biome simulations^33^ with a database of chronometrically dated eastern African MSA stone tool assemblages^11^ to refine the characterisation of MSA environments, map their extent across the landscape, and explore the role of environmental variability in structuring patterns of behaviour.

## Results

### Middle and Late Pleistocene climates of MSA occupations

**Figure 2:**
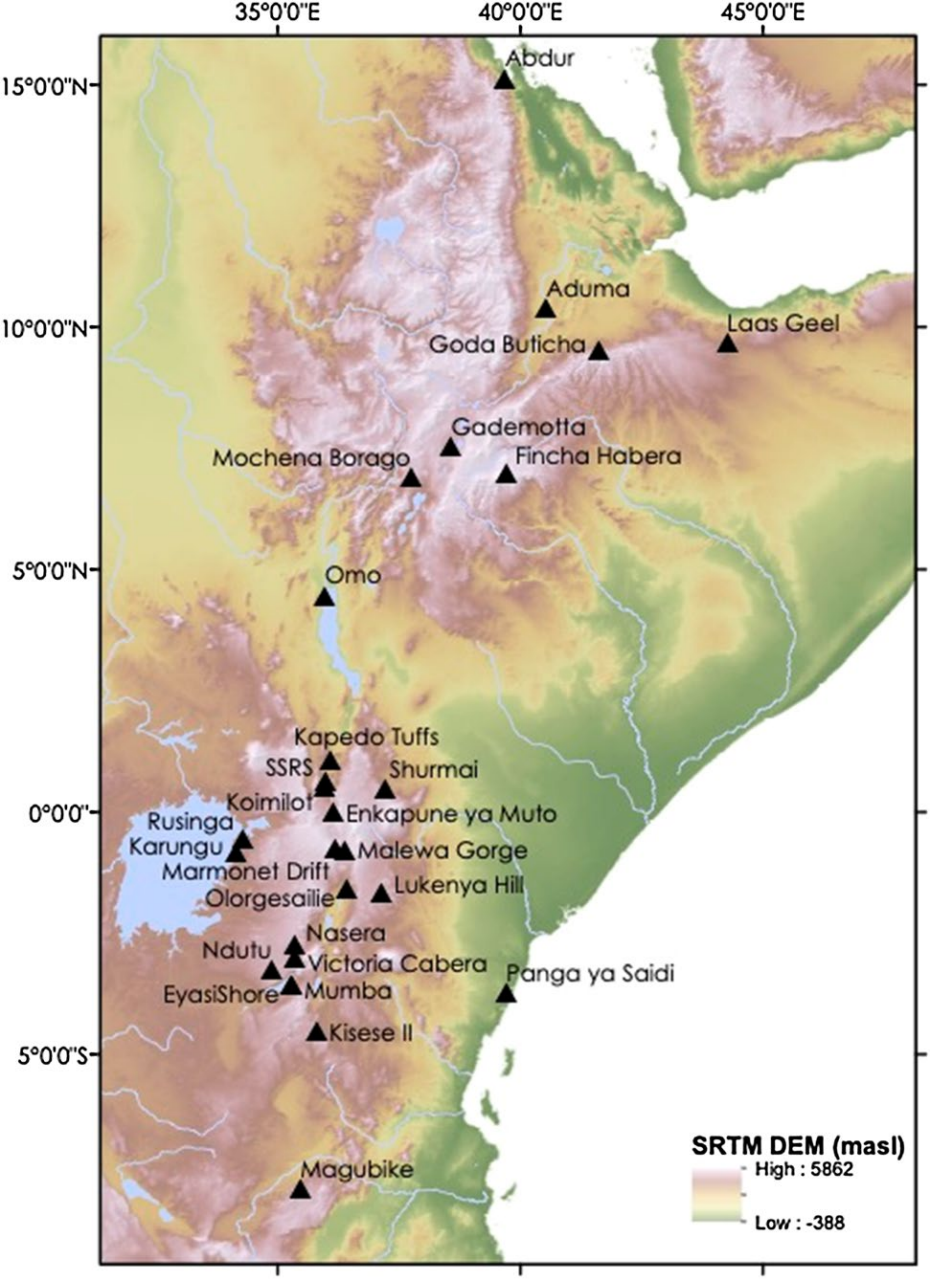
Distribution of the eastern African Middle Stone Age occupations studied. This map was created in ArcGIS 10.5 using an SRTM (NASA).

We first examined MSA occupations (n = 84, Fig. 2) spanning the Middle to Late Pleistocene using simulated climate data (see Methods). We extracted mean annual temperature (bio01) and total annual precipitation (bio12) values from the climate model^33^ within a 50 km radii, centred on the occupation’s mid-age date range rounded to the nearest 1000 year (kyr) time slice, to characterise environments across the wider logistical landscape (following Blinkhorn and Grove^10,11^). The climatic conditions for each occupation can be found in Supplementary Table S1 and are illustrated in Fig. 1.

We found that average temperatures at eastern African MSA occupations varied between 9 °C and 25 °C, with 59 occupations falling within the 68% confidence interval of 14–23 °C. The warmest environments occupied were found in coastal regions, such as Abdur along the Red Sea coast of modern-day Eritrea (25 °C) and Panga Ya Saidi situated on the Kenyan coast (24 °C), as well as in the Lower Omo Valley of southwestern Ethiopia (24–23 °C). These hot environments were inhabited during MIS 5 and MIS 7. On the other hand, the coldest environments inhabited were at high altitude, at Fincha Habera in the Bale Mountains of southern Ethiopia (9–10 °C) and at Kenyan Rift Valley occupations of Marmonet Drift (10–14 °C) and Enkapune ya Muto (13 °C), most of which date to MIS 3. Average precipitation levels experienced by Middle to Late Pleistocene MSA populations in eastern Africa ranged between 396 and 1593 mm, with 59 occupations falling within the 68% confidence interval of 620-1150 mm, corresponding to the precipitation bracket of sub-humid landscapes. The wettest habitats were located on islands within and along the shore of Lake Victoria at Rusinga Nyamtia (1593 mm) and Karungu (1374-1499 mm) in MIS 3 and 5, as well as within the Ethiopian Rift Valley at Gademotta (1368 mm), the Ethiopian Highlands at Mochena Borango (1270-1297 mm), and the Kenyan Rift Valley at Marmonet Drift (1173-1368 mm) in MIS 3, 5 and 7. On the other hand, the driest occupations occurred at Laas Geel in Somaliland during MIS 3 (396 mm) as well as within the Lower Omo Valley (534-582 mm) during MIS 5 and 7.

### Classifying biomes and ecotones at MSA occupations

**Figure 3:**
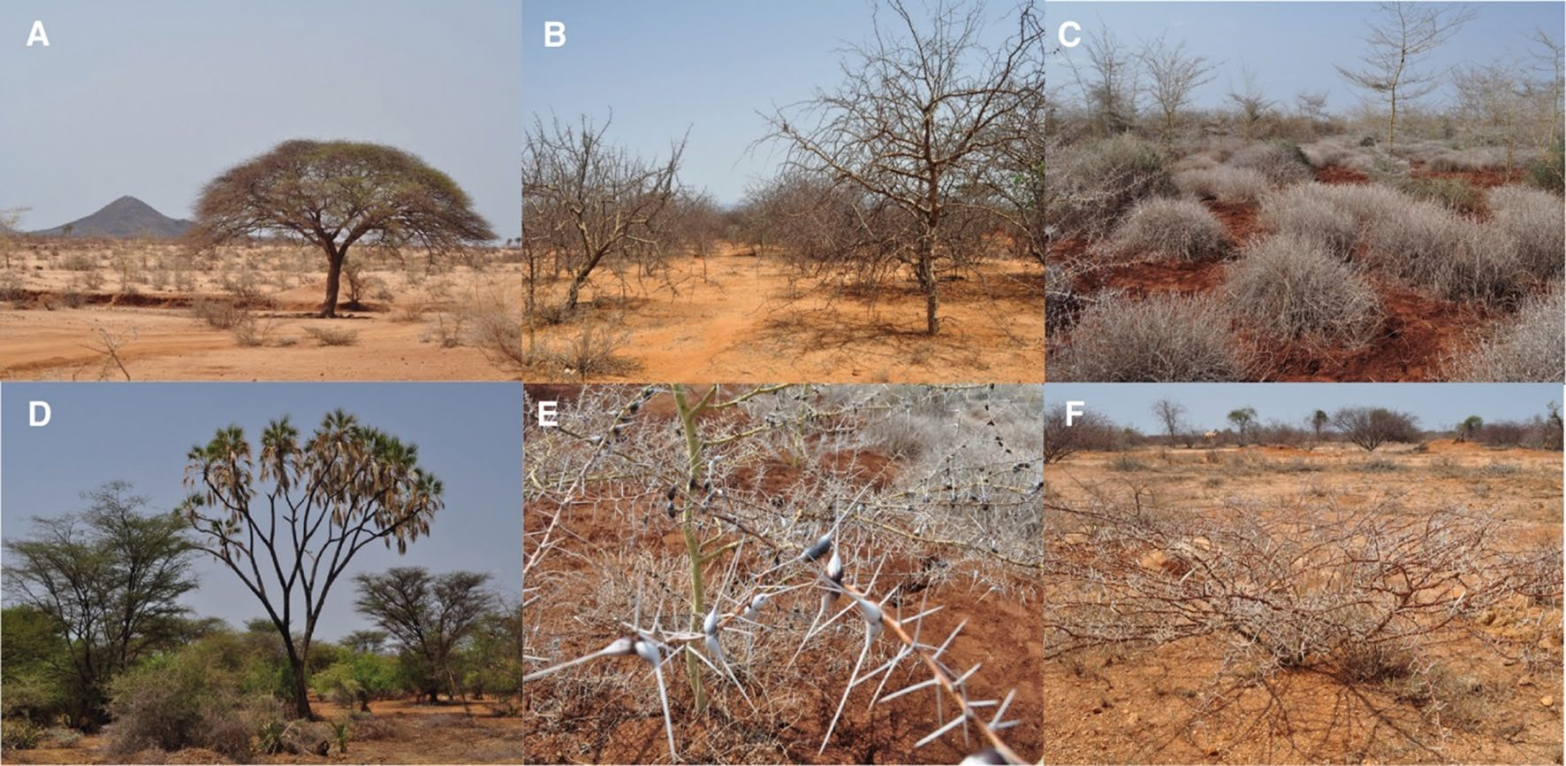
Examples of xerophytic shrubland environments in modern eastern Africa, including typical species (sp.). (**A**) *Acacia tortilis* (**B**) *Commiphora *sp. (**C**) *Acacia *sp. and *Duosperma eremophilum.* (**D**) *Hyphaene compressa*, *Acacia* sp., *Salvadora persica*, Cyperacea and *Lawsonia inermis* (**E**) *Acacia *sp. and *Duosperma eremophilum*, (**F**) *Acacia tortilis* (background: *Commiphora *sp. Capparaceae sp*. Tephrosia sp*. and *Indigofera spinosa*).

We then used the modelled biome dataset (biome4output)^33^ to classify the local ecology of each MSA occupation within a 50 km radius. We found that 38% of the occupations (n = 32) had access to only tropical xerophytic shrubland within their logistical landscape (see Fig. 3. for modern examples of this biome), and a further 42% with this biome among others within a 50 km radius (n = 35). Tropical xerophytic shrubland was persistently occupied throughout the Middle to Late Pleistocene (Fig. 1), and whilst it was the most prevalent biome type available, representing 61.9% of the biomes present during occupational phases across the region (Supplementary Fig. S2 and Table S2), eastern African MSA adaptive systems were likely specialised for engagement with tropical xerophytic shrubland, and its modulation may therefore have influenced patterns of Middle to Late Pleistocene human distribution. Nonetheless, the proportion of occupations with access to tropical xerophytic shrubland was significantly higher using a 2-sample proportion test than the proportion of the biome available across the region throughout MSA occupational phases (Z-value = 3.38, p-value = 0.0007; Supplementary Table S2), suggesting preferential occupation of tropical xerophytic shrubland and emphasising it as an important ecosystem for MSA populations.

In total, 57% of the occupations had a logistical landscape falling on the boundary between multiple biomes (n = 48; Supplementary Table S1). The majority of these ecotonal sites are situated between ‘open’ and ‘closed’ biome types, supporting the assertion of Basell^9^ that access to wooded ecologies was vital for MSA populations. Forest biomes made up relatively low proportions of the available environments available throughout the Middle to Late Pleistocene; however, importantly, we found the proportions of forest biomes occupied by MSA occupations to be significantly higher than would be expected based on the prevalence of these biomes, especially in MIS 3 and MIS 7 (see Supplementary Fig. S2 and Table S2), supporting the contention that MSA hominins preferred the rarer habitats that were near to woods and forests. The most common ecotone occupied during the eastern African MSA was that between tropical xerophytic shrubland and temperate conifer forest, which is seen as far north as Goda Buticha in southeastern Ethiopia, and as far south as Mumba in Tanzania. However, the region to the east of Lake Victoria shows the most intense occupation of this ecotone, the boundary of which fluctuates through time and space (Supplementary Table S1).

We found that MIS 7 saw the preferential occupation of closed ecotones between temperate conifer forest and warm mixed forest, as well as tropical xerophytic shrubland and associated ecotones which are generally occupied throughout the period. MIS 5 saw a slight increase in habitat diversity, though expansions primarily involved the tracking of tropical xerophytic shrubland environments (as shown by all occupations in MIS 5 having access to this biome within 50 km) with exposure to new ecotones occurring at the peripheries. This can be seen at occupations distributed widely across the region; for example, certain occupations at Omo would have involved engagement with deserts alongside tropical xerophytic shrubland, whereas some MSA populations at Panga Ya Saidi had access to tropical deciduous forest and tropical savannah environments within their logistical landscape. MIS 3 saw the greatest variety in the ecologies occupied, where expansions can be seen into new and previously uninhabited environments, such as steppe tundra and warm mixed forest, with a distinct emphasis on temperate conifer forest rather than tropical xerophytic shrubland. Importantly, a chi-square test revealed that the relative proportions of biomes in the region do not differ significantly between the Marine Isotope Stages (χ^2^ = 9.07, p-value = 0.99), strongly suggesting that variation in the environments occupied through time reflects a shift in preference as opposed to fluctuation in the underlying ecology (see Supplementary Table S2).

### Characterising MSA environments throughout the Middle to Late Pleistocene

**Figure 4:**
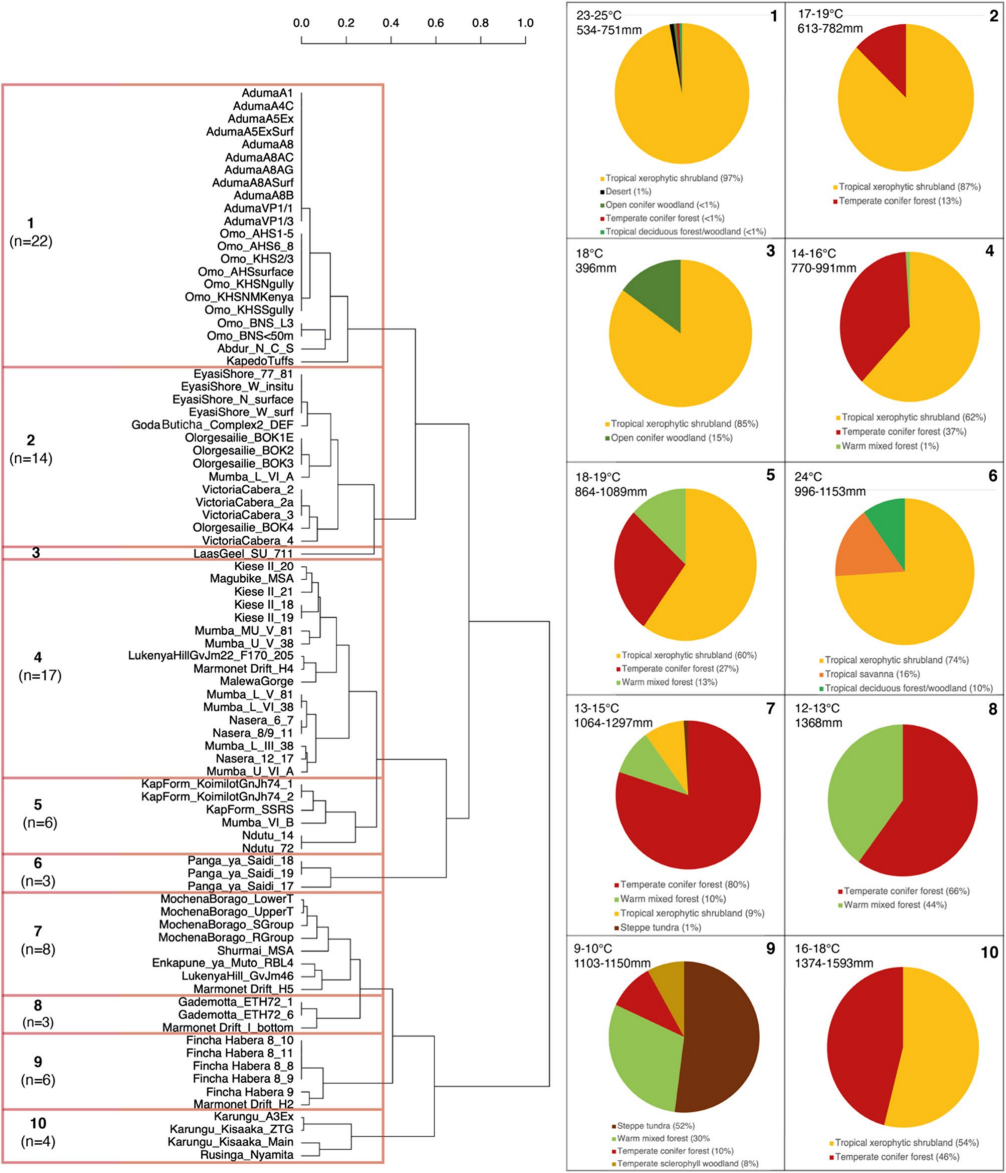
Hierarchical clustering of the occupations according to mean annual temperature and total annual precipitation. K means clustering identified ten clusters as the optimal division of the dendrogram, which have been highlighted here as well as the range of environmental conditions occupied by each cluster and the percentage of cells within 50 km of that biome for all occupations within that cluster.

We used cluster analyses to group the occupations based on their climatic values to assess patterns in habitat choice. To do this, we scaled and combined the temperature and precipitation data and employed an automated clustering algorithm (the average silhouette method) to ascertain the optimal number (k) of clusters in the data. The algorithm found ten clusters to represent the best division of the data (Fig. 4, Supplementary Fig. S1).

Most of the occupations (n = 45) fall within warm to temperate sub-humid clusters (2,4,5 and 7) with a broad temperature range of 13–19 °C and a precipitation range of 613-1297 mm. These clusters are dominated by tropical xerophytic shrubland and temperate conifer forest environments and their ecotones. We found that only two clusters (8,9) did not include occupations with access to tropical xerophytic shrubland, indicating that this biome was present across a large portion of the MSA climatic range, except at the coldest extreme. We found that the coldest cluster, cluster 9 (temperature range 9–10 °C), was the most ecotonal, with all occupations situated at high altitude where populations would have had access to steppe tundra, temperate conifer forest, temperate sclerophyll woodland and warm mixed forest, the complex topography allowing diverse biomes to appear closer together than is usually possible^34^. Extremely humid occupations from around Lake Victoria (Karungu and Rusinga Nyamita) formed cluster 10 (1374-1593 mm precipitation). These occupations have moderate temperatures (16–18 °C) and occupy an ecotone between tropical xerophytic shrubland and temperate conifer forest. Panga Ya Saidi and Laas Geel form their own respective clusters (3 and 6) due to their distinctively hot temperatures; however, at Panga Ya Saidi, this is coupled with moist sub-humid conditions and a diverse tropical environment (24 °C, 996-1153 mm), whereas Laas Geel possesses the lowest annual precipitation of all the occupations (18 °C, 396 mm), making its hot-dry environment unique for the eastern African MSA. However, the occupation at Laas Geel falls within the tropical xerophytic shrubland biome, with access to some open conifer woodland within 50 km, suggesting that whilst occupying a climatic extreme, this distinct habitat represents an extension of the types of environments that eastern African MSA populations were already well-adapted to.

### Phased habitability models

We used the precipitation and temperature data from the occupations as the parameters to produce phased ‘habitability’ models for the more abundantly populated interglacial phases of the MSA, demonstrating the extent of the landscape that experienced comparable climatic settings to occupations dated within that period. The climatic range produced by each phased subset was projected throughout every 1000-year time interval for that MIS, and then the percentage of ‘habitable’ cells (i.e., cells that remain within that climatic range) was calculated to identify areas that were persistently habitable, as well as the geographic range and temporal scope of impersistent habitable landscapes.

**Figures 5:**
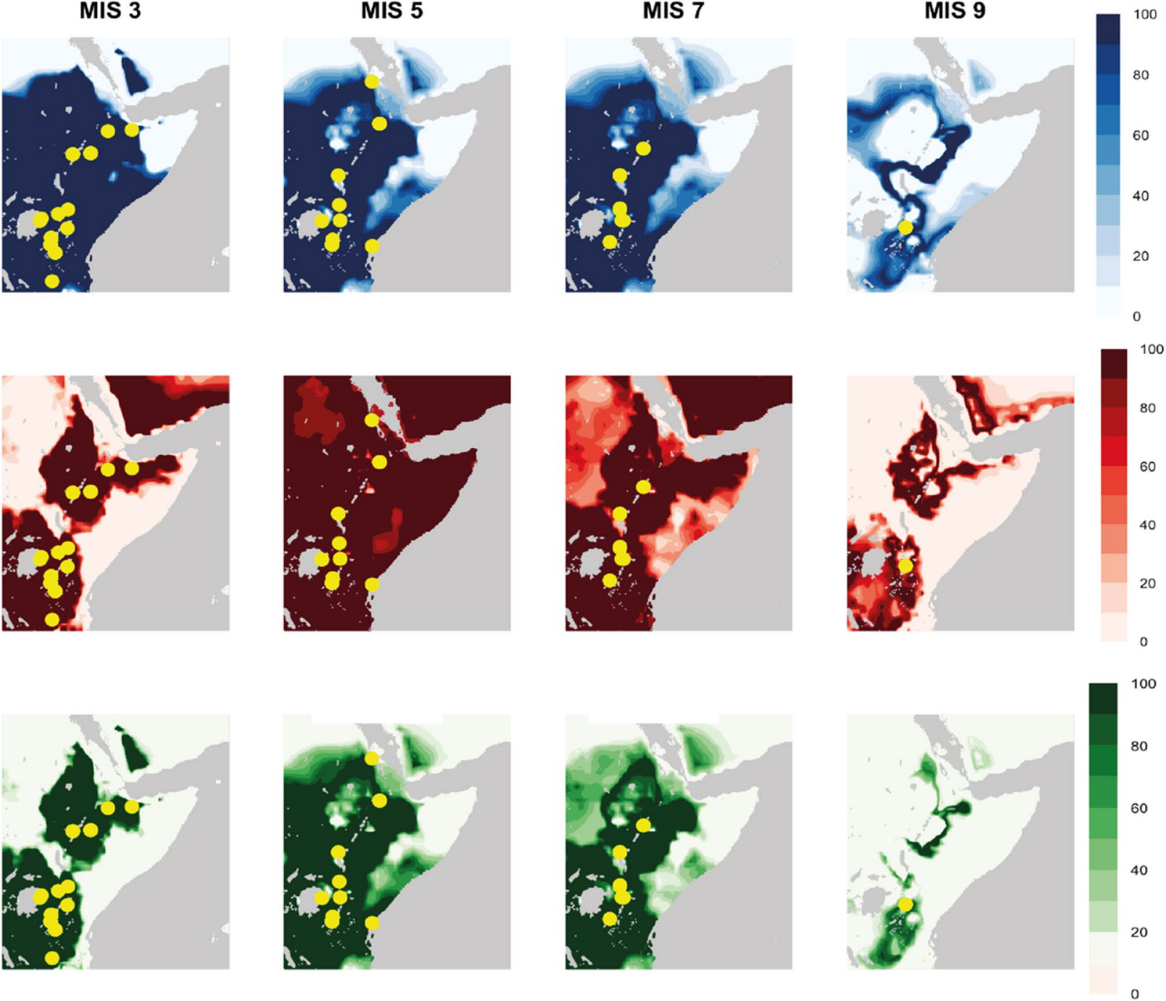
Mean annual temperature (top), total annual precipitation (middle) and combined (bottom) phased models of habitability, demonstrating the percentage of time intervals (1000 years per interval) that remain within the climatic range of the occupations dated to that Marine Isotope Stage (MIS). The palaeocoastline has been estimated based on the predicted mean sea-level for each MIS.

Figure 5 demonstrates the temperature, precipitation, and combined habitability models for each phase. MIS 9 shows the most limited habitable zone out of the interglacial phases, however the lower number of occupations available to construct the distribution likely has impacted the construction of the models. MIS 7 marks a period of expansion, with the region surrounding Lake Victoria and the Eastern Rift Valley Lakes and the Ethiopian Highlands showing the most persistent habitability across the region. For temperature, large areas of the Horn and modern-day Sudan show less persistent habitability (*ca.* 40–50% cells falling within the temperature range of 12–23 °C seen at MIS 7 occupations), with pockets of unsuitability along the coast of the Baab el Mandeb and the border between modern-day Ethiopian and Somalia. However, arid zones of the southern Sahara are completely uninhabitable in terms of precipitation (0% of cells fall within the precipitation range of 582-1368 mm at MIS 7 occupations), as is the tip of the Horn. Precipitation is thus the limiting factor when considering habitability for MIS 7, as the area deemed habitable in terms of precipitation is more geographically restricted than that derived from temperature. MIS 5 sees the largest increase in habitable area for temperature, with all cells showing temperature values within the MIS 5 occupation range of 13–25 °C for at least 60% of the period. Precipitation habitability, that we considered here to be ranging between 554-1385 mm, is however more fragmented, with pockets of uninhabitability forming around the northeast edge of Lake Victoria, in the region to the south of Lake Tana, and within modern-day Tanzania. Like MIS 7, this means that habitability is limited by precipitation in MIS 5. However, the habitability models for MIS 3 demonstrates the opposite pattern. Temperature habitability, defined as between 9–19 °C by the sites dating to MIS 3, shows the most restricted distribution of all the models, with habitable areas concentrated to the areas around Lake Victoria and the Ethiopian highlands, which are linked towards the southeast of Lake Turkana. Yet, MIS 3 shows the most persistent and widely distributed zone of habitability for precipitation, where much of eastern Africa, except towards the Sahara and the very tip of the Horn of Africa, remains persistently within the range of precipitation values experienced by MIS3 occupations (396-1593 mm). Overall, these models propose that interglacial MSA occupations, especially in MIS 5, may have been much more spatially diverse than presently known, however we note that these distributions are based purely on climatic data and ignore the potential effects of volcanic eruptions and subsequent ashfalls that have also been argued to have conditioned habitability in this region^9^.

**Figures 6:**
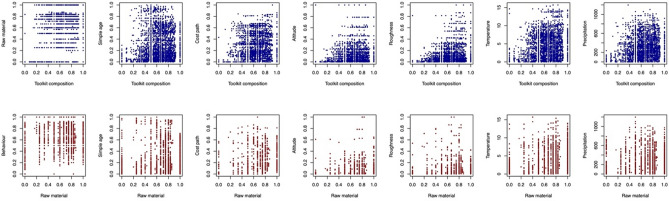
Scatterplots of the Mantel test results (Table 1, Supplementary Table S4–S5) between the pairwise distance matrix of toolkit composition (top) and raw material use (bottom) and the other distances matrices excluding the two binary variables, site type and method.

### Exploring the relationship between climate and Middle Stone Age occupations

We then examined the extent to which patterns of variability in stone tool assemblage composition and raw material use correlated with environmental conditions within a 50 km radius at the mid-age of occupation of each assemblage, as well as a suite of other variables recorded by Blinkhorn and Grove^11^ (see Methods and Supplementary Methods S1 details). Figure 6 demonstrates the relationships between these variables and toolkit composition and raw material use, revealed using simple Mantel tests (Table 1 and Supplementary Table S4–S5). We found that MSA assemblage composition was correlated with differences in both mean annual temperature (adj.* p* = 0.001; Table 1) and total annual precipitation (adj.* p* = 0.003; Table 1), and raw material use also shows statistically significant relationships with both mean annual temperature (adj. * p* = 0.001; Table 1) and total annual precipitation (adj. * p* = 0.003; Table 1). With the use of Pleistocene climate models at high temporal resolutions, these results refine the findings of Blinkhorn and Grove^11^, which relied on comparisons of the climatic extremes of the LGM and LIG.

**Table 1:** Simple Mantel test results for the effects of precipitation and temperature on toolkit composition and raw material. Statistical significance highlighted at *p* < 0.05 (*) or *p* < 0.01 (**). The Benjamini–Hochberg procedure was used to adjust p values.

	Toolkit composition			Raw material		
	Coefficient	*p*	Adj. *p*	Coefficient	*p*	Adj. *p*
Precipitation	0.1972	0.001**	0.003**	0.1587	0.001**	0.003**
Temperature	0.2144	0.001**	0.003**	0.1532	0.001**	0.001**

We next employed multiple matrix regressions to resolve independent, significant correlations between differences in either stone tool assemblage composition or raw material use, and differences across the different variables. Table 2 demonstrates that four variables exhibit independent significant effects on toolkit composition (F = 68.0, multiple R^2^ = 0.15, *p* = 0.001)—raw material use, roughness, site type and precipitation. In the multiple matrix regression for raw material (Table 2), five variables were found to have independent effects (F = 54.8, multiple R^2^ = 0.12,* p* = 0.001) – toolkit composition, cost path, roughness and simple age, with precipitation being very close to significant. In each instance, these results complement earlier findings, but the use of highly resolved climate models illustrates the independent and significant relationship between MSA behaviour and precipitation that was not evident from previous analyses that were reliant on models for climatic extremes^11^. This highlights the benefit of using highly chronologically resolved model data to more accurately characterise the climatic parameters of MSA occupations in eastern Africa and to reveal the relationship between environmental and behavioural variability.

**Table 2:** Multiple matrix regression results for toolkit composition and raw material. Descriptions of each variable can be found in the Supplementary Methods. Statistical significance highlighted at *p* < 0.05 (*) or *p* < 0.01 (**).

	Toolkit composition		Raw material	
	Coefficient	p	Coefficient	*p*
Toolkit composition	NA	NA	0.3119	0.001**
Raw material	0.1483	0.001**	NA	NA
Method	0.009	0.698	0.0351	0.192
Site type	0.0328	0.018*	− 0.0061	0.7
Simple Age	0.0184	0.668	0.149	0.007**
Cost path	0.0172	0.669	0.1991	0.001**
Altitude	− 0.09	0.473	0.2206	0.106
Roughness	0.2645	0.004**	− 0.2286	0.017*
Temperature	0.0673	0.298	− 0.0108	0.896
Precipitation	0.111	0.015*	0.1124	0.053

## Discussion

Using high-resolution modelled climatic data, we have conducted more sophisticated analyses of the environments inhabited by MSA-making populations than was previously possible, bridging former disconnects between archaeological and climatic records within a spatiotemporally explicit framework. Figure 1 highlights the challenge of generalising from proxy core records which we have overcome through the parameterisation of temperature and precipitation with simulated models, revealing interpretable patterns in terms of MSA environments. Our analyses have extended previous work applying models of the LGM and LIG to represent climatic extremes^10,11^ and importantly have revealed the climatic bracket of eastern African MSA occupations to around 9–25 °C mean annual temperature and 396-1593 mm total annual precipitation. MSA occupations are more prevalent during interglacial phases (MIS 3, 5, and 7) and we found that populations during these periods inhabited more diverse and widely distributed environments. Most occupations are located within or with access to tropical xerophytic shrubland, and usually are found within proximity to at least one forest or woodland biome, even though these environments only make up a small proportion of those available throughout the Middle to Late Pleistocene. Using spatiotemporally explicit models we also established that precipitation levels experienced by MSA populations had an impact on both behavioural variability and the use of raw materials, which we explore further here using penalised logistic regression.

Our results highlight spatial and diachronic trends in the environments inhabited by eastern African MSA populations, which are demonstrated by Fig. 1, 3 and Supplementary Table S1. MIS 9 and 8 saw occupations that were typical for the eastern African MSA in terms of temperature, precipitation, altitude, and biomes inhabited. During MIS 7, there was a dramatic increase in the environments occupied, spanning much of the climatic range of the later interglacial phases with emphasis on forest/woodland ecotones and tropical xerophytic shrubland. MIS 6 saw the occupation of a cold, high-altitude environment; however, the phase is only represented by a single occupation, Marmonet Drift_H2, with a large date range so we do not place too much emphasis on its mid-age climatic placement. MIS 5 was marked by another phase of expansion, though with limited presence in woodland habitats and typically warmer, drier and low altitude occupations. MIS 5 has been proposed to represent a transitional point within the MSA stone tool record, due to the augmentation of MSA toolkits with new combinations of stone tools and the colonisation of different landscapes into the Late Pleistocene^9,10,13^. Engagement with these new environments likely occurred at the edge of the logistical landscape, with many eastern African MSA occupations falling on ecotonal boundaries between tropical xerophytic shrubland and previously unoccupied biome types. An example of this is found with the youngest MSA occupation at Panga Ya Saidi, which is placed at a unique tropical ecotone^35^. MSA occupation of Panga Ya Saidi at the end of MIS 5 is rapidly followed by cultural change, with the appearance of the Later Stone Age here occurring earlier than anywhere else in the region^35^. New constellations of tools within MIS 5 toolkits suggest corresponding changes in behaviour previously unseen in the MSA^10^, which perhaps may have been key for these expansions beyond tropical xerophytic shrubland. Together, this suggests that certain MSA toolkits may have led to adaptation, as opposed to adaptations being a pre-requisite for expansion, with transient ecotonal habitats requiring the adoption of a range of survival strategies and potentially mediating interaction between different groups across different environmental contexts^2,35,36^. Occupations dated to MIS 4 show a climatic range close to that of MIS 3, where we see a push into higher precipitation woodland habitats with a limited change in temperature and altitude. Habitability modelling for MIS 3 suggests that temperature, rather than precipitation as seen during earlier interglacial periods, constrained expansions into new landscapes, with populations occupying cooler landscapes than seen previously during the MSA, specifically within mountainous settings. There is some discussion about whether MIS 3 can be considered a true interglacial due to its erratic nature^37^ however, it does include some very warm (albeit short-lived) periods, and thus mountain ranges may have offered refuges when lower-altitude landscapes were especially arid^38^. Moreover, unpredictable climates have been found to favour the evolution of ‘generalist’ strategies and behavioural plasticity^39^, perhaps explaining why there appears to be a notable extension of the precipitation regime inhabited during MIS 3, with the occupation of both extreme dry and wet environments.

Eastern African MSA occupations are primarily located within tropical xerophytic shrubland, characterised by arid-adapted species such as that from the *Acacia and Commiphora* genera. Whilst it is the most abundant biome across modern eastern Africa as well as the Middle to Late Pleistocene (see Supplementary Fig. S2 and Table S2), the intense occupation of tropical xerophytic shrubland and its associated ecotones suggests that eastern African MSA adaptive strategies were centred around engaging with subsistence resources associated with this biome. Our results also confirm the importance of access to wooded ecotones for sustaining MSA populations^9^, with the most common ecotone occurring between temperate conifer forest and tropical xerophytic shrubland, distributed widely across the region though most concentrated towards the eastern edge of Lake Victoria and the associated region. Today, Lake Victoria sits on the junction between central African rainforests and savanna habitats to the east, forming an important boundary for large mammal^40^ and human^41^ populations. Our phased models (Fig. 5) propose that the region to the east of Lake Victoria, including the Eastern African Rift Valley, would have seen sustained and persistent occupation throughout the Middle to Late Pleistocene in the face of climatic fluctuation, implicating it as a potential refugium for hominins in eastern Africa. The availability of freshwater provided by surrounding rivers and springs^42^ and the complex topography of the Rift Valley^31,43^ may also have helped buffer against the strongest effects of climate change in the area^18^. Occupying refugia likely required minimal cultural adaptation to environmental change, which could explain why assemblages from within the Lake Victoria basin show distinct differences from the general eastern African MSA^44^. Moreover, Lake Victoria, which is relatively young but estimated to exist by 500–400 kya^45^, is the source of the White Nile, and therefore its refugial position could also have interesting implications for dispersals from the region out of the continent^46^. Together, this highlights the potential role of refugia in structuring MSA cultural and biological diversity^3,4^, laying the critical foundations for later human evolution, with microhabitat variability in refugial zones likely being a key component in creating resource-rich landscapes^32,47^.

Beyond simply characterising MSA environments and ecologies, our use of matrix correlations presents an important means to examine the extent to which climatic features influenced behaviour, here characterised by stone tool assemblage composition. Our multiple matrix regression results demonstrate that precipitation has a significant, independent effect on toolkit composition; further examination of the presences and absences of individual technologies via binary logistic regression shows statistically significant decreases in the probability of backed microliths, borers, centripetal technology, platform cores, and scrapers occurring in assemblages as precipitation levels increase, after controlling for all other variables in the analysis (see Supplementary Methods S2 and Supplementary Table S3). Some of these tool types have been found to be significant indicators of either Later Stone Age (backed pieces) or MSA (scrapers) toolkits^48,49^, perhaps linking changes in precipitation to the development of the LSA. Roughness also has a significant impact on toolkit composition, with further analyses demonstrating that the probability of backed microliths and points, both components of projectile technology associated with hunting of grassland species in flatter landscapes^50^ as well as Levallois blades and flakes decreases as the energetic demands of the environment increase. Further analysis of the site type variable demonstrates that it has significant effects on the probability of backed microliths, bipolar technology, and Levallois flakes appearing in assemblages, with all these technologies occurring more frequently in cave or rock shelter sites. Finally, there are numerous independent effects of the use of particular raw materials on the presence of individual technologies in the assemblages, as summarised in Supplementary Table S3. Whilst precipitation has a close to significant effect on raw material use, independent explanations of variability are presented by cost path, roughness, simple age and toolkit composition. This constellation of geographic variables suggests a complex combination of spatial autocorrelation, likely grounded in the uneven availability of raw materials across eastern Africa as well as their accessibility in different landscape settings, such as mountains. Overall, these results demonstrate a complex interaction between the environment and MSA behaviour, with the energetic requirements of the physical landscape and local precipitation regimes likely requiring different constellations of tools from the MSA toolkit.

## Conclusion

The MSA record of eastern Africa is fundamental for the exploration of ideas surrounding the role of climate change and the environment in recent human evolution and dispersals within and beyond the continent. Our application of high-resolution paleoenvironmental reconstructions has enabled a previously impossible characterisation of the ecosystems inhabited by early human populations at this scale, with existing site-based paleoenvironmental reconstructions being very useful but difficult to integrate and translate into a comprehensive framework for the region. Whilst the application of high-resolution climatic simulations to archaeological data is inherently limited by the uncertainty surrounding radiometric dates – indeed, future work should focus on developing methods that address the effects of dating error on conclusions derived from climatic models (see^51^) – our results are important for demonstrating the environmental conditions inhabited throughout the eastern African MSA, with levels of diversity far beyond that typically assumed by classic habitat-specific hypotheses for human evolution^52–55^. By establishing the role of shifting environmental conditions and ecological boundaries on the distribution and variability of dated MSA assemblages, our work also helps illuminate some of the processes that shaped behavioural diversity during this key period, such as that MSA toolkits likely facilitated expansions into diverse environments, adding further complexity to within-region migration than environment tracking^56^. Understanding this complex interaction between population dynamics, dispersals, cultural evolution and the environment is also key to moving beyond simplistic single-origin models in favour of more complex reticulate models for recent human evolution^3,4^.

## Materials and methods

We employed here simulated climate and biome reconstructions for the past 800 ka^33^. Mean annual temperature (bio01), total annual precipitation (bio12) and biome (biomeoutput04) were extracted from the model for 1000 year time slices and stored in a raster stack using the ‘raster’ R package^57^. We then selected the time slices corresponding to the mid-age of each assemblage (the mid-point between the minimum and maximum date range of the occupation), rounded to the nearest 1000 years to match the resolution of the climate data (see Supplementary Table S1). Each time slice was cropped according to the predicted sea level for that period, using global sea-level change data^58^ and a bathymetry model^59^ (see Supplementary Methods S3).

We integrated the climate model with an archaeological dataset reported in Blinkhorn and Grove^11^ which comprises presence/absence data of 16 tool types and 8 raw material types for eastern African MSA assemblages, as well as other typological characteristics, such as site type and method of excavation (see Supplementary Methods S1 for further description).

### Refining the climatic parameters

We extracted temperature and precipitation values^33^ in 50 km radius buffers around the coordinates of each occupation from its mid-age time slice, representing the home range sizes of hunter-gatherer populations^60^ and regional patterns of local raw material procurement^6,61–63^. In order to better characterise patterns of landscape variability across this range, we downscaled model datasets from 30’ to 2.5’ by resampling the data using bilinear interpolation, following examination of alternate methods (see Supplementary Methods S4). We calculated the mean and standard deviation of the values across the 50 km buffer for each occupation.

### Cluster analyses

Dissimilarity matrices for the temperature and precipitation data were calculated using the Euclidean distance. Hierarchical clustering of the dissimilarity matrices was then employed to examine grouping of occupations according to temperature and precipitation combined in equal combinations to characterise environments, following Blinkhorn and Grove^10^. Cluster analysis iteratively merges cases into a group using a measure of closeness (i.e., the opposite of dissimilarity). We applied the average silhouette method, which uses k-means clustering to automatically determine the optimal number of clusters from the data. The average silhouette method evaluates the quality of clustering by determining how well each occupation lies within its cluster, producing a measure of how similar an occupation is to its own cluster relative to other clusters. We created a dendrogram of the occupations (Fig. 4) which was cut according to the k-means output (k = 10) to define our clusters.

### Biome classification

To classify the biomes occupied by each occupation, we used the modelled biome dataset^32^. To increase the resolution of the data to 2.5’, we resampled the raster layers using the nearest neighbour method, which is most suitable for categorical data. We extracted the biome type for each assemblage location at its mid-age and then recorded the number of cells (each representative of 4.63 km) assigned to each biome within a 50 km radius around each occupation. To evaluate whether the proportion of biomes inhabited by MSA populations was significantly different to the proportion of biomes avaliable across the landscape, we used 2-sample proportion tests. We also used a chi-square test to investigate whether biome proportions changed through time to test whether changes in habitat are due to preference shifts throughout the MSA or a reflection of the underlying ecologies available.

### Phased climate suitability models

We used the temperature and precipitation values from within a 50 km of each occupation to produce phased models of ‘habitability’. To do this, we subsetted the occupations according to MIS, and produced a series of climatic ranges based on the inhabited temperature and precipitation values during that period. We then calculated the percentage of time slices within each MIS that fall within the climatic range of occupations dated to that period. This produced phased models mapping the areas of the landscape that would have been comparable to those inhabited during that MIS, indicating the degree of habitat stability over time.

### Mantel tests and multiple matrix regression

To explore the relationships between toolkit diversity, raw material choice and environments, we used simple Mantel tests and multiple matrix regression, the null hypothesis for which assumes that there is no correlation between two distance matrices (e.g., toolkit composition and temperature), factoring in the impact of additional variables (e.g., raw material) for multiple matrix regressions. Positive correlation in dissimilarity between two distance matrices, like toolkit composition and temperature, would suggest that occupations experiencing similar temperatures share more similar constellations of tool types, whereas a negative correlation would suggest that as dissimilarity increases in one variable, it decreases in another.

As described in Blinkhorn and Grove^11^, the additional distance matrices used as variables that may explain variation in MSA toolkits for multiple matrix regression are site type, method, simple age (mid-age), cost path (a measure of physical distance), altitude within 50 km and roughness within 50 km (calculated as amount of energy needed to move across the logistical landscape). A description of how each distance measure was calculated can be found in the Supplementary Methods S4, following Blinkhorn and Grove^11^. Simple Mantel tests were conducted using the ‘vegan’ R package^64^, employing 999 permutations to obtain p-values. The Benjamini–Hochberg procedure was used to adjust *p* values. Multiple matrix regression was performed with 999 permutations using the ‘phytools’ package in R^65^.

Additional binary logistic regressions using penalized maximum likelihood were conducted to determine the effects of significant predictors on individual technologies using the ‘brglm’ R package^66^ (see Supplementary Methods S2). Unlike our initial correlation statistics that use a single distance measure to represent archaeological diversity, binary logistic regression is based on the presences and absences of certain technologies, with each regression controlled for the effects of all other variables, except cost path which is only available as a distance matrix and thus inappropriate for logistic regression analysis. Supplementary Table S3 demonstrates the positive and negative significant effects of independent variables on each tool type.

## Supplementary Information

Supplementary Information.

## Data Availability

The datasets used in this study are all freely available^11,33,50,59^. Code for the analyses can be found at https://osf.io/nxfer/ and were presented as Supplementary Material for the purposes of the review process.
