# Supplementary material for: A spatiotemporally explicit paleoenvironmental framework for the Middle Stone Age of eastern Africa
Source: Sci Rep. 2022 Mar 7;12:3689. doi: 10.1038/s41598-022-07742-y (PMC8901736; doi:10.1038/s41598-022-07742-y)
Supplement: Supplementary file 1 — Supplementary Information. [file 41598_2022_7742_MOESM1_ESM.docx]

**A spatiotemporally explicit paleoenvironmental framework for the Middle Stone Age of eastern Africa**

Timbrell, L., Grove, M., Manica, A., Rucina, S. and Blinkhorn., J.

**Supplementary Online Materials**

Supplementary Table S1. Summary of the Middle Stone Age occupations studied. Mean and standard deviation of mean annual temperature, total annual precipitation, and altitude are calculated across 50km for each occupation. Minimum, maximum, and mid-point age is derived from Blinkhorn and Grove [1], where radiometric dates for each assemblage collected from original references. Biome classification was extracted at the site coordinates and ecotones expressed as the percentage of cells per biome within 50km at each occupation.

| Assemblage | Temperature (℃) | Precipitation (mm) | Altitude (meters above sea level) | Minimum age (kya) | Maximum age (kya) | Mid-age (kya) | Biome classification | Ecotones |
| --- | --- | --- | --- | --- | --- | --- | --- | --- |
| Goda Buticha_Complex2_DEF | 19 (2) | 742 (90) | 1478 (564) | 22 | 70 | 46 | Temperate conifer forest | Temperate conifer forest (65%), Tropical xerophytic shrubland (35%) |
| Mumba_L_III_38 | 16 (0) | 800 (25) | 1514 (353) | 33 | 40 | 37 | Tropical xerophytic shrubland | Temperate conifer forest (36%), Tropical xerophytic shrubland (64%) |
| Mumba_L_V_81 | 16 (0) | 797 (28) | 1514 (353) | 52 | 61 | 57 | Tropical xerophytic shrubland | Temperate conifer forest (36%), Tropical xerophytic shrubland (64%) |
| Mumba_L_VI_38 | 16 (0) | 797 (28) | 1514 (353) | 52 | 61 | 57 | Tropical xerophytic shrubland | Temperate conifer forest (36%), Tropical xerophytic shrubland (64%) |
| Mumba_L_VI_A | 18 (0) | 782 (33) | 1514 (353) | 86 | 115 | 101 | Tropical xerophytic shrubland | Tropical xerophytic shrubland (100%) |
| Mumba_MU_V_81 | 16 (0) | 917 (33) | 1514 (353) | 47 | 55 | 51 | Tropical xerophytic shrubland | Temperate conifer forest (38%), Tropical xerophytic shrubland (62%) |
| Mumba_U_V_38 | 15 (0) | 949 (28) | 1514 (353) | 44 | 53 | 49 | Tropical xerophytic shrubland | Temperate conifer forest (38%), Tropical xerophytic shrubland (62%) |
| Mumba_U_VI_A | 15 (0) | 804 (24) | 1514 (353) | 57 | 69 | 63 | Tropical xerophytic shrubland | Temperate conifer forest (38%), Tropical xerophytic shrubland (62%)) |
| Mumba_VI_B | 19 (0) | 864 (34) | 1514 (353) | 86 | 153 | 120 | Tropical xerophytic shrubland | Tropical xerophytic shrubland (100%) |
| Panga_ya_Saidi_17 | 24 (0) | 1152 (45) | 143 (84) | 65 | 80 | 73 | Tropical savanna | Tropical deciduous forest/woodland (28%), Tropical savanna (48%), Tropical xerophytic shrubland (24%) |
| Panga_ya_Saidi_18 | 24 (0) | 996 (42) | 143 (84) | 67 | 86 | 77 | Tropical xerophytic shrubland | Tropical xerophytic shrubland (100%) |
| Panga_ya_Saidi_19 | 24 (0) | 996 (42) | 143 (84) | 67 | 86 | 77 | Tropical xerophytic shrubland | Tropical xerophytic shrubland (100%) |
| Enkapune_ya_Muto_RBL4 | 13 (1) | 1118 (69) | 1960 (386) | 43 | 45 | 45 | Warm mixed forest | Temperate conifer forest (37%), Warm mixed forest (63%) |
| Fincha Habera 8_10 | 9 (1) | 1150 (21) | 2712 (613) | 27 | 34 | 31 | Steppe tundra | Steppe tundra (204), Temperate conifer forest (31), Temperate sclerophyll woodland (15), Warm mixed forest (120) |
| Fincha Habera 8_11 | 9.0 (1) | 1150 (21) | 2712 (613) | 27 | 34 | 31 | Steppe tundra | Steppe tundra (55%), Temperate conifer forest (9%), Temperate sclerophyll woodland (4%), Warm mixed forest (32%) |
| Fincha Habera 8_8 | 9.0 (0.8) | 1150 (21) | 2712 (613) | 27 | 34 | 31 | Steppe tundra | Steppe tundra (55%), Temperate conifer forest (9%), Temperate sclerophyll woodland (4%), Warm mixed forest (32%) |
| Fincha Habera 8_9 | 9.0 (0.8) | 1150 (21) | 2712 (613) | 27 | 34 | 31 | Steppe tundra | Steppe tundra (55%), Temperate conifer forest (9%), Temperate sclerophyll woodland (4%), Warm mixed forest (32%) |
| Fincha Habera 9 | 10 (1) | 1144 (21) | 2712 (613) | 33 | 42 | 38 | Steppe tundra | Steppe tundra (55%), Temperate conifer forest (9%), Temperate sclerophyll woodland (4%), Warm mixed forest (32%)) |
| Kiese II_18 | 16 (0) | 865 (22) | 1397 (232) | 38 | 39 | 39 | Tropical xerophytic shrubland | Temperate conifer forest (2%), Tropical xerophytic shrubland (98%) |
| Kiese II_19 | 16 (0) | 865 (22) | 1397 (232) | 31 | 46 | 39 | Tropical xerophytic shrubland | Temperate conifer forest (2%), Tropical xerophytic shrubland (98%) |
| Kiese II_20 | 16 (0) | 928 (21) | 1397 (232) | 42 | 45 | 44 | Tropical xerophytic shrubland | Temperate conifer forest (2%), Tropical xerophytic shrubland (98%)) |
| Kiese II_21 | 16 (0) | 911 (20) | 1397 (232) | 43 | 46 | 45 | Tropical xerophytic shrubland | Temperate conifer forest (2%), Tropical xerophytic shrubland (98%)) |
| LaasGeel_SU_711 | 18 (1) | 396 (56) | 1113 (189) | 40 | 42 | 42 | Tropical xerophytic shrubland | Open conifer woodland (15%), Tropical xerophytic shrubland (85%) |
| LukenyaHillGvJm22_F170_205 | 14 (1) | 934 (67) | 1547 (149) | 25 | 32 | 29 | Temperate conifer forest | Temperate conifer forest (332), Tropical xerophytic shrubland (34) |
| Magubike_MSA | 16 (1) | 955 (86) | 1365 (394) | 35 | 48 | 42 | Temperate conifer forest | Temperate conifer forest (88%), Tropical xerophytic shrubland (12%) |
| MochenaBorago_LowerT | 15 (0) | 1282 (19) | 1638 (345) | 49 | 50 | 50 | Temperate conifer forest | Temperate conifer forest (100%) |
| MochenaBorago_RGroup | 14 (0) | 1297 (19) | 1638 (345) | 36 | 43 | 40 | Temperate conifer forest | Temperate conifer forest (98%), Warm mixed forest (2%) |
| MochenaBorago_SGroup | 15 (0) | 1270 (19) | 1638 (345) | 43 | 46 | 45 | Temperate conifer forest | Temperate conifer forest (100%) |
| MochenaBorago_UpperT | 15 (0) | 1274 (19) | 1638 (345) | 45 | 49 | 48 | Temperate conifer forest | Temperate conifer forest (100%) |
| Nasera_12_17 | 16 (0) | 778 (41) | 1710 (289) | 53 | 58 | 56 | Temperate conifer forest | Temperate conifer forest (71%), Tropical xerophytic shrubland (29%) |
| Nasera_6_7 | 16 (0) | 770 (41) | 1710 (289) | 50 | 58 | 54 | Tropical xerophytic shrubland | Temperate conifer forest (16%), Tropical xerophytic shrubland (86%) |
| Nasera_8/9_11 | 16 (0) | 770 (41) | 1710 (289) | 50 | 58 | 54 | Tropical xerophytic shrubland | Temperate conifer forest (16%), Tropical xerophytic shrubland (86%) |
| Shurmai_MSA | 15 (2) | 1165 (109) | 1443 (374) | 39 | 50 | 45 | Temperate conifer forest | Steppe tundra (2%), Temperate conifer forest (55%), Tropical xerophytic shrubland (43%) |
| Abdur_N_C_S | 25 (2) | 658 (99) | 773 (798) | 118 | 132 | 125 | Tropical xerophytic shrubland | Open conifer woodland (18%), Tropical xerophytic shrubland (82%) |
| AdumaA1 | 23 (1) | 620 (75) | 723 (166) | 80 | 100 | 90 | Tropical xerophytic shrubland | Tropical xerophytic shrubland (100%) |
| AdumaA4C | 23 (1) | 620 (75) | 723 (166) | 80 | 100 | 90 | Tropical xerophytic shrubland | Tropical xerophytic shrubland (100%) |
| AdumaA5Ex | 23 (1) | 620 (75) | 723 (166) | 80 | 100 | 90 | Tropical xerophytic shrubland | Tropical xerophytic shrubland (100%) |
| AdumaA5ExSurf | 23 (1) | 620 (75) | 723 (166) | 80 | 100 | 90 | Tropical xerophytic shrubland | Tropical xerophytic shrubland (100%) |
| AdumaA8 | 23 (1) | 620 (75) | 723 (166) | 80 | 100 | 90 | Tropical xerophytic shrubland | Tropical xerophytic shrubland (100%) |
| AdumaA8AC | 23 (1) | 620 (75) | 723 (166) | 80 | 100 | 90 | Tropical xerophytic shrubland | Tropical xerophytic shrubland (100%) |
| AdumaA8AG | 23 (1) | 620 (75) | 723 (166) | 80 | 100 | 90 | Tropical xerophytic shrubland | Tropical xerophytic shrubland (100%) |
| AdumaA8ASurf | 23 (1) | 620 (75) | 723 (166) | 80 | 100 | 90 | Tropical xerophytic shrubland | Tropical xerophytic shrubland (100%) |
| AdumaA8B | 23 (1) | 620 (75) | 723 (166) | 80 | 100 | 90 | Tropical xerophytic shrubland | Tropical xerophytic shrubland (100%) |
| EyasiShore_77_81 | 19 (0) | 713 (19) | 1508 (356) | 91 | 132 | 112 | Tropical xerophytic shrubland | Tropical xerophytic shrubland (100%) |
| EyasiShore_W_insitu | 19 (0) | 713 (19) | 1508 (356) | 91 | 132 | 112 | Tropical xerophytic shrubland | Tropical xerophytic shrubland (100%) |
| Gademotta_ETH72_1 | 13 (1) | 1368 (48) | 1898 (316) | 172 | 274 | 223 | Warm mixed forest | Temperate conifer forest (46%), Warm mixed forest (54%) |
| Gademotta_ETH72_6 | 13 (1) | 1368 (48) | 1898 (316) | 172 | 274 | 223 | Warm mixed forest | Temperate conifer forest (46%), Warm mixed forest (54%) |
| KapForm_KoimilotGnJh74_1 | 18 (1) | 977 (86) | 1428 (369) | 198 | 237 | 218 | Temperate conifer forest | Temperate conifer forest (61%), Warm mixed forest (39%) |
| KapForm_KoimilotGnJh74_2 | 18 (1) | 977 (86) | 1428 (369) | 198 | 237 | 218 | Temperate conifer forest | Temperate conifer forest (61%), Warm mixed forest (39%) |
| KapForm_SSRS | 19 (1) | 947 (83) | 1319 (335) | 198 | 237 | 218 | Tropical xerophytic shrubland | Temperate conifer forest (42%), Tropical xerophytic shrubland (58%) |
| Karungu_A3Ex | 18 (1) | 1385 (108) | 1254 (125) | 42 | 115 | 79 | Tropical xerophytic shrubland | Temperate conifer forest (17%), Tropical xerophytic shrubland (83%) |
| LukenyaHill_GvJm46 | 13 (1) | 1064 (63) | 1547 (149) | 21 | 27 | 24 | Temperate conifer forest | Temperate conifer forest (17%), Tropical xerophytic shrubland (83%) |
| Marmonet Drift_H2 | 10 (1) | 1103 (110) | 2215 (341) | 89 | 205 | 147 | Temperate sclerophyll woodland | Steppe tundra (28%), Temperate conifer forest (20%), Temperate sclerophyll woodland (43%), Warm mixed forest (9%) |
| Marmonet Drift_H4 | 14 (1) | 937 (114) | 2215 (341) | 90 | 130 | 110 | Temperate conifer forest | Temperate conifer forest (64%), Tropical xerophytic shrubland (18%), Warm mixed forest (18%) |
| Marmonet Drift_H5 | 13 (1) | 1173 (113) | 2215 (341) | 90 | 98 | 94 | Temperate conifer forest | Temperate conifer forest (68%), Tropical xerophytic shrubland (20%), Warm mixed forest (17%) |
| Marmonet Drift_I_bottom | 12 (1) | 1368 (113) | 2215 (341) | 205 | 257 | 231 | Warm mixed forest | Temperate conifer forest (40%), Warm mixed forest (60%) |
| Olorgesailie_BOK1E | 17 (2) | 770 (108) | 1360 (381) | 295 | 320 | 308 | Tropical xerophytic shrubland | Temperate conifer forest (16%), Tropical xerophytic shrubland (84%) |
| Olorgesailie_BOK2 | 17 (2) | 770 (108) | 1360 (381) | 295 | 320 | 308 | Tropical xerophytic shrubland | Temperate conifer forest (16%), Tropical xerophytic shrubland (84%) |
| Olorgesailie_BOK3 | 17 (2) | 770 (108) | 1360 (381) | 295 | 320 | 308 | Tropical xerophytic shrubland | Temperate conifer forest (16%), Tropical xerophytic shrubland (84%) |
| Olorgesailie_BOK4 | 17 (2) | 675 (112) | 1360 (381) | 288 | 301 | 295 | Tropical xerophytic shrubland | Temperate conifer forest (64%), Tropical xerophytic shrubland (36%) |
| Omo_AHS1-5 | 23 (0) | 582 (48) | 450 (118) | 193 | 201 | 197 | Tropical xerophytic shrubland | Tropical xerophytic shrubland (100%) |
| Omo_AHS6_8 | 23 (0) | 582 (48) | 450 (118) | 193 | 201 | 197 | Tropical xerophytic shrubland | Tropical xerophytic shrubland (100%) |
| Omo_BNS_L3 | 24 (0) | 534 (48) | 450 (118) | 96 | 111 | 104 | Tropical xerophytic shrubland | Desert (13%), Tropical xerophytic shrubland (87%) |
| Omo_KHS2/3 | 23 (0) | 582 (48) | 450 (118) | 193 | 201 | 197 | Tropical xerophytic shrubland | Tropical xerophytic shrubland (100%) |
| VictoriaCabera_2 | 17 (0) | 657 (25) | 1765 (349) | 70 | 91 | 81 | Tropical xerophytic shrubland | Tropical xerophytic shrubland (100%) |
| VictoriaCabera_2a | 17 (0) | 657 (25) | 1765 (349) | 70 | 91 | 81 | Tropical xerophytic shrubland | Tropical xerophytic shrubland (100%) |
| VictoriaCabera_3 | 17 (0) | 657 (25) | 1765 (349) | 70 | 91 | 81 | Tropical xerophytic shrubland | Tropical xerophytic shrubland (100%) |
| VictoriaCabera_4 | 18 (0) | 613 (23) | 1765 (349) | 79 | 91 | 86 | Tropical xerophytic shrubland | Tropical xerophytic shrubland (100%) |
| AdumaVP1/1 | 23 (1) | 620 (75) | 723 (166) | 80 | 100 | 90 | Tropical xerophytic shrubland | Tropical xerophytic shrubland (100%) |
| AdumaVP1/3 | 23 (1) | 620 (75) | 723 (166) | 80 | 100 | 90 | Tropical xerophytic shrubland | Tropical xerophytic shrubland (100%) |
| EyasiShore_N_surface | 19 (0) | 713 (19) | 1508 (356) | 91 | 132 | 112 | Tropical xerophytic shrubland | Tropical xerophytic shrubland (100%) |
| EyasiShore_W_surf | 19 (0) | 713 (19) | 1508 (356) | 91 | 132 | 112 | Tropical xerophytic shrubland | Tropical xerophytic shrubland (100%) |
| KapedoTuffs | 23 (1) | 751 (91) | 1106 (267) | 120 | 135 | 128 | Tropical xerophytic shrubland | Temperate conifer forest (2%), Tropical deciduous forest/woodland (15%), Tropical xerophytic shrubland (83%) |
| Karungu_Kisaaka_Main | 16 (1) | 1499 (93) | 1231 (116) | 42 | 56 | 49 | Temperate conifer forest | Temperate conifer forest (70%), Tropical xerophytic shrubland (30%) |
| Karungu_Kisaaka_ZTG | 18 (1) | 1374 (97) | 1230 (116) | 42 | 115 | 79 | Tropical xerophytic shrubland | Temperate conifer forest (12%), Tropical xerophytic shrubland (88%) |
| MalewaGorge | 15 (1) | 991 (182) | 2240 (388) | 240 | 240 | 240 | Temperate conifer forest | Temperate conifer forest (74%), Tropical xerophytic shrubland (26%) |
| Ndutu_14 | 17 (0) | 1089 (37) | 1527 (209) | 220 | 240 | 230 | Tropical xerophytic shrubland | Tropical xerophytic shrubland (100%) |
| Ndutu_72 | 17 (0) | 1089 (37) | 1527 (209) | 220 | 240 | 230 | Tropical xerophytic shrubland | Tropical xerophytic shrubland (100%) |
| Omo_AHSsurface | 23 (0) | 582 (48) | 450 (118) | 193 | 201 | 197 | Tropical xerophytic shrubland | Tropical xerophytic shrubland (100%) |
| Omo_BNS<50m | 24 (0) | 534 (48) | 450 (118) | 96 | 111 | 104 | Tropical xerophytic shrubland | Desert (13%), Tropical xerophytic shrubland (87%) |
| Omo_KHSNgully | 23 (0) | 582 (48) | 450 (118) | 193 | 201 | 197 | Tropical xerophytic shrubland | Tropical xerophytic shrubland (100%) |
| Omo_KHSNMKenya | 23 (0) | 582 (48) | 450 (118) | 193 | 201 | 197 | Tropical xerophytic shrubland | Tropical xerophytic shrubland (100%) |
| Omo_KHSSgully | 23 (0) | 582 (48) | 450 (118) | 193 | 201 | 197 | Tropical xerophytic shrubland | Tropical xerophytic shrubland (100%) |
| Rusinga_Nyamita | 16 (1) | 1593 (134) | 1226 (114) | 42 | 56 | 49 | Temperate conifer forest | Temperate conifer forest (100%) |


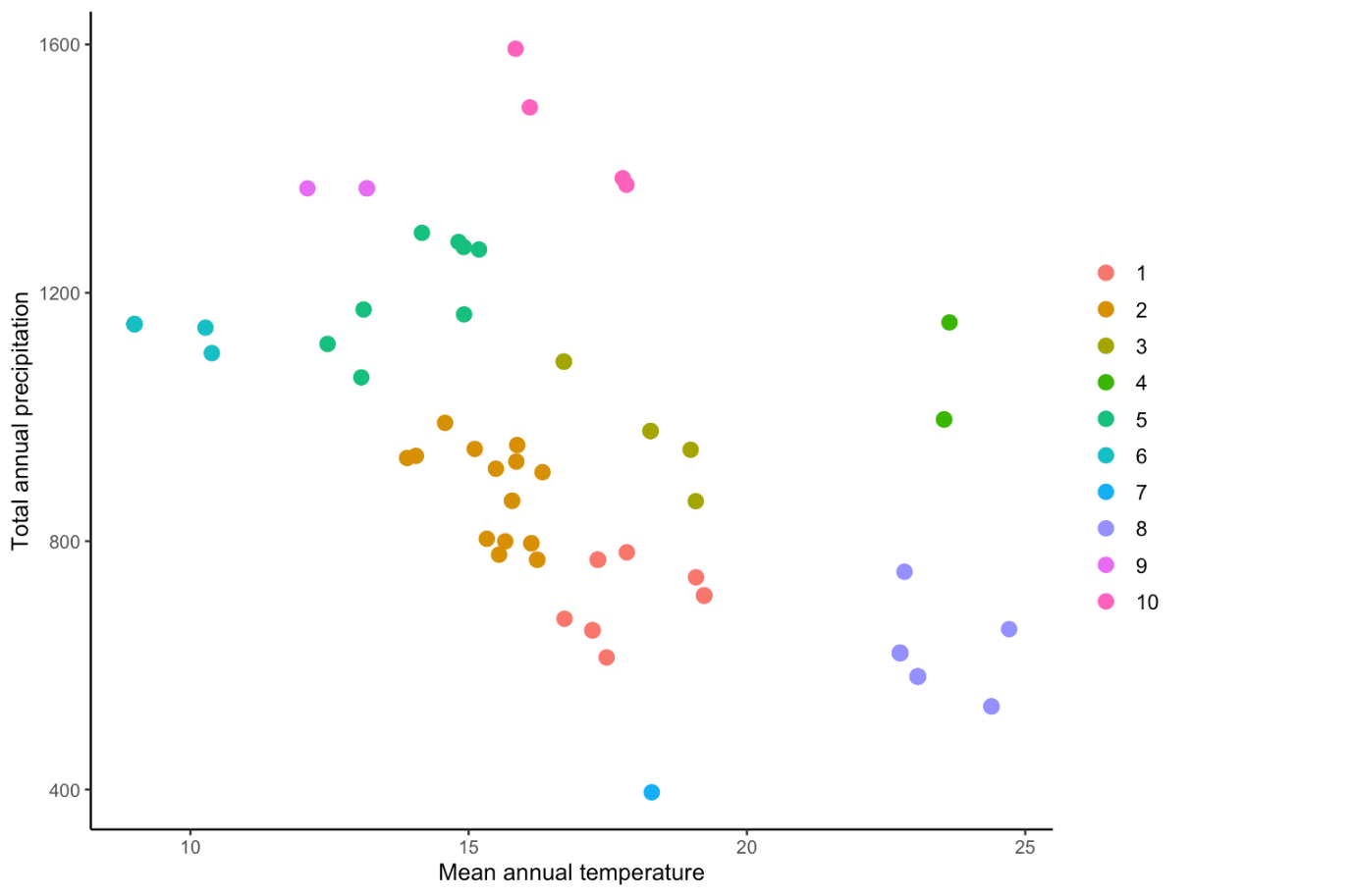
Supplementary Figure S1. The k-means (n = 10) clusters plotted against mean annual temperature (℃) and total annual precipitation (mm). The average silhouette method identified that ten was the optimal division of the data, and this value was used to cut the resulting dendrogram of the sites.


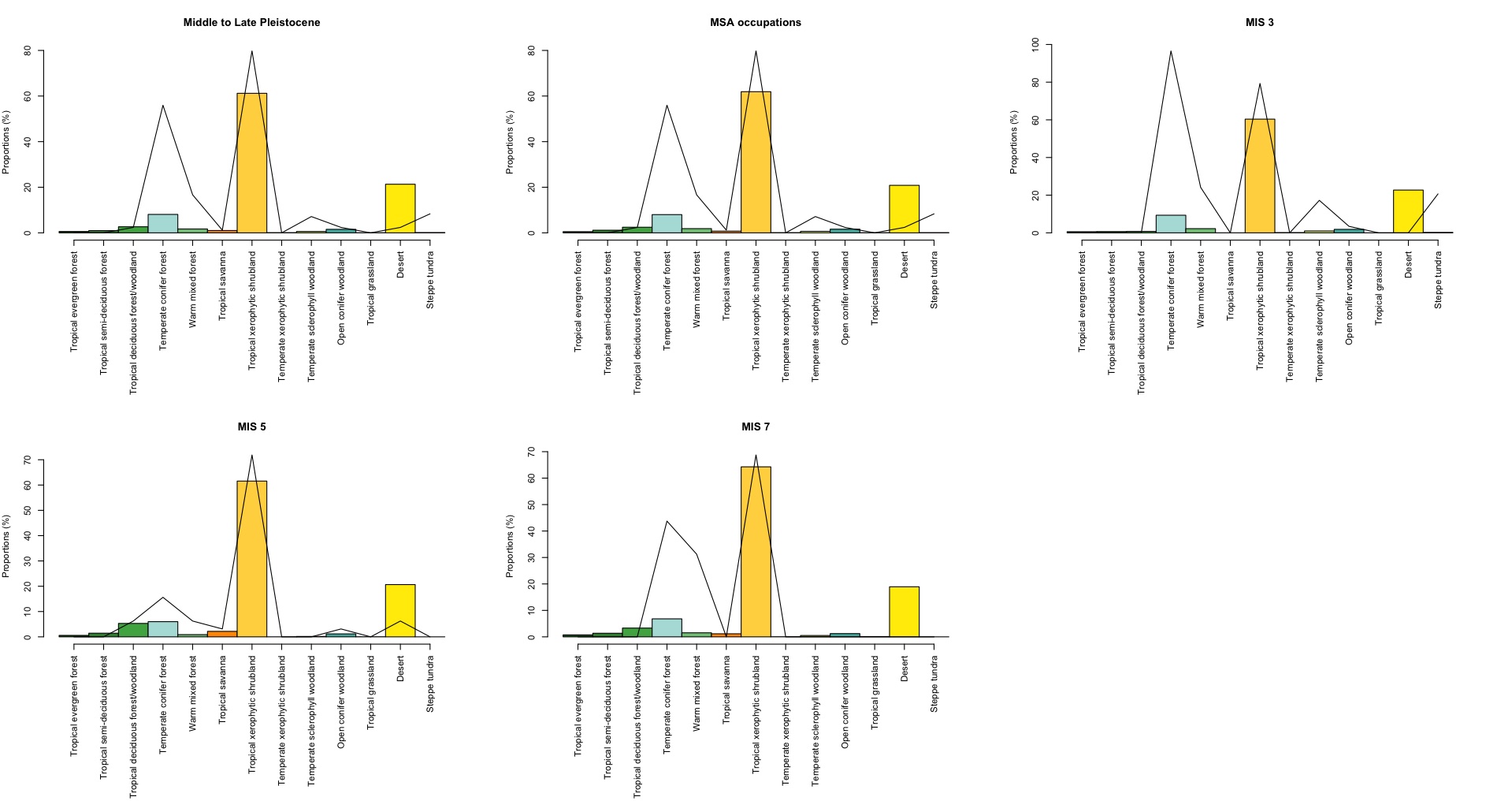
Supplementary Figure S2. Proportion (expressed as a percentage) of biomes available across eastern African during the Middle to Late Pleistocene, for full date range of the assemblages (21-320 kya), MSA occupational phases and Marine Isotope Stage (MIS) 3, MIS 5 and MIS 7 (bars) with the percentage of biomes available at eastern African MSA occupations through time (lines).

Supplementary Table S2. Proportions of biomes available across eastern Africa (P1) throughout the Middle to Late Pleistocene (full date range of the assemblages from 21-320ka), MSA occupational phases, and Marine Isotope Stage (MIS) 3, MIS 5 and MIS 7, compared to the proportions of biomes available at MSA occupations (P2). Grey cells represent the biomes available in eastern Africa that were not inhabited by MSA populations during that period.

| Biome | Full date range | | MSA occupations | | MIS 3 | | MIS 5 | | MIS 7 | |
| --- | --- | --- | --- | --- | --- | --- | --- | --- | --- | --- |
|  | P1 | P2 | P1 | P2 | P1 | P2 | P1 | P2 | P1 | P2 |
| Tropical evergreen forest | 0.006 |  | 0.005 |  | 0.60 |  | 0.58 |  | 0.74 |  |
| Tropical semi-deciduous forest | 0.010 |  | 0.011 |  | 0.68 |  | 1.45 |  | 1.36 |  |
| Tropical deciduous forest/woodland | 0.027 | 0.024 | 0.025 | 0.024 | 0.78 |  | 0.053 | 0.063 | 3.31 |  |
| Temperate conifer forest | 0.081 | 0.560 | 0.080 | 0.560 | 0.094 | 0.966 | 0.060 | 0.156 | 0.068 | 0.438 |
| Warm mixed forest | 0.017 | 0.167 | 0.019 | 0.167 | 0.022 | 0.241 | 0.009 | 0.063 | 0.015 | 0.313 |
| Tropical savanna | 0.012 | 0.012 | 0.008 | 0.012 | 0.02 |  | 0.022 | 0.031 | 1.16 |  |
| Tropical xerophytic shrubland | 0.612 | 0.798 | 0.619 | 0.798 | 0.603 | 0.793 | 0.616 | 1.000 | 0.643 | 0.688 |
| Temperate xerophytic shrubland | 0.001 |  | 0.001 |  | 0.15 |  | 0.01 |  | 0.05 |  |
| Temperate sclerophyll woodland | 0.006 | 0.071 | 0.006 | 0.071 | 0.010 | 0.172 | 0.16 |  | 0.51 |  |
| Open conifer woodland | 0.015 | 0.024 | 0.016 | 0.024 | 0.019 | 0.034 | 0.012 | 0.031 | 1.22 |  |
| Tropical grassland | 0.001 |  | 0.001 |  | 0.02 |  | 0.05 |  | 0.11 |  |
| Desert | 0.213 | 0.024 | 0.208 | 0.024 | 0.227 |  | 0.207 | 0.063 | 18.92 |  |
| Steppe tundra | 0.001 | 0.083 | 0.001 | 0.083 | 0.002 | 0.207 | 0.00 |  | 0.00 |  |

Supplementary Table S3. Results from two sample proportion tests, comparing the proportions of biomes available across the region and the biomes occupied during that period (see Supplementary Table S2 and Figure S2). Z values and p-values are reported here to demonstrate the strength of effects and where there are significant differences at p < 0.05 (*) or p < 0.01 (**). Grey cells represent the biomes available in eastern Africa that were not inhabited by MSA hominins during that period.

| Biome | Full date range | | MSA occupations | | MIS 3 | | MIS 5 | | MIS 7 | |
| --- | --- | --- | --- | --- | --- | --- | --- | --- | --- | --- |
|  | Z | p | Z | p | Z | p | Z | p | Z | p |
| Tropical deciduous forest/woodland | 0.000 | 1.000 | 0.000 | 1 |  |  | 0.000 | 1 |  |  |
| Temperate conifer forest | 15.882 | <0.0001** | 15.974 | <0.0001** | 15.764 | <0.0001** | 1.918 | 0.055 | 5.368 | <0.0001** |
| Warm mixed forest | 10.186 | <0.0001** | 9.631 | <0.0001** | 7.336 | <0.0001** | 2.294 | 0.022* | 8.676 | <0.0001** |
| Tropical savanna | 0.000 | 1.000 | 0.000 | 1 |  |  | 0.000 | 1 |  |  |
| Tropical xerophytic shrubland | 3.38 | 0.0007** | 3.257 | 0.0004** | 1.895 | 0.058 | 3.574 | 0.0004** | 0.113 | 0.91 |
| Temperate sclerophyll woodland | 7.025 | <0.0001** | 6.642 | <0.0001** | 7.843 | <0.0001** |  |  |  |  |
| Open conifer woodland | 0.184 | 0.8542 | 0.131 | 0.8958 | 0.000 | 1 | 0.204 | 0.6838 |  |  |
| Desert | -4.104 | <0.0001** | -4.030 | <0.0001** |  |  | -1.794 | 0.073 |  |  |
| Steppe tundra | 18.626 | <0.0001** | 21.497 | <0.0001** | 20.208 | <0.0001** |  |  |  |  |

**Supplementary Methods:**

S1. Calculation of distance matrices

Following Blinkhorn and Grove [1], we produced a series of distance matrices to explore effects of age, distance in space, and geographic and environmental characteristics of the landscapes surrounding them on toolkit composition and raw material use. Here, we provide a basic description of each variable include in the matrix correlations, however further description and the data can be found in Blinkhorn and Grove [1].

Toolkit composition is representative by the presence/absence of 16 artefact categories within the assemblage. These are backed pieces/microliths, bipolar technology, blade technology, borers, burins, centripetal technology, core tools, denticulates, Levallois blade technology, Levallois flake technology, Levallois point technology, notched pieces, platform cores, point technology, retouched bifacial tools and scrapers. Raw material use was recorded as the presence/absences of 8 categories: cherts, quartz, obsidians, basalts, metamorphics, crypto-crystalline silica (CCS), other igneous and other sedimentary. Site types involved the designation between open-air sites or rock shelters and caves. Method of site investigation was recorded as either excavation or survey. We used the mid-age of each assemblage as a simple estimation of the date of each assemblage. Maximum and minimum dates were ascertained from the literature based on the errors of the dating, and the median was taken to represent the mid-age.

Cost path:

To represent the physical distance between sites, we calculated the cost path as this is a more representative measure than the Euclidean or geodesic distance. Cost of movement over heterogeneous landscapes was determined to predict the fastest routes between the eastern African sites. Cost path analyses calculate which route across a rough topographic landscape joins two points (sites) with the lowest accumulated cost. In this sense, whilst the shortest distance between two sites is a straight line (the Euclidean distance), it may be much faster, and therefore less costly, to walk around a mountain even if the distance is further.

We applied Tobler’s Hiking Function which is often used cost-analyses to estimate the maximum hiking speed ($s$) given the slope of the terrain ($m$) [2]. It is preferred over just the slope as it accounts for anisotropy (the direction of movement has an impact on the cost) and nonlinear cost estimation (the cost of travel can change far more rapidly than the linear change from one input to the next). The maximum speed of off-path hiking (in km/h) is calculated as:

$s=6e^{-3.5\left| m+0.05 \right|}$

Tobler’s Hiking function is not symmetric around 0, and this is because humans tend to walk fastest on gently downward slopes ($m = -0.05$), where they can walk faster than on flat terrain ($m = 0$). To compute a transition layer using the Hiking Function, we calculated the slope $m$ of the terrain from the altitude $z$ and the distance between cell centres $d$ of each DEM for each pair of cells $i$ and $j$. This was performed using the gdistance package in R [3], with major water bodies masked from the analysis.

$m_{ij}=\left( z_{j}-z_{i} \right)/d_{ij}$

The slope ($m$) was then used to calculate the travel time $T$ in hours of moving between cells of the DEM using the reciprocal of Tobler Hiking Function:

$T=0.6e^{3.5\left| m+0.05 \right|}$

Finally, a correction procedure was employed to consider the distance between cell centres, as when travelling with the same speed, a diagonal connection between cells takes longer to cross than a straight connection.

Altitude and roughness at 50km:

We sampled from the slope ($m$) rasters at a 50km radius around each site to ascertain the mean and standard deviation of altitude of the logistical landscape each assemblage was situated in. ﻿We then transformed the slope $(m$) raster into values of energy expenditure (Joules per metre per second for an average 60kg person) following Minetti *et al.* [4]. This formula accounts for the fact that there are different energetic costs ($cw$) associated with moving on flat ground vs 10° slopes compared to 10° and 20° slopes, despite the difference in slope being the same.

$cw=60\cdot\left( \left( 280.5m^{5} \right)-\left( 58.7m^{4} \right)-\left( 76.8m^{3} \right)+\left( 51.9m^{2} \right)+\left( 19.6m \right)+2.5 \right)$

We sampled from the resulting roughness rasters at a 50km radius around each occupation and took the mean and standard deviation.

S2. Logistic regressions of individual technologies on significant predictors

Following the multiple matrix regressions, we conducted further analyses on significant predictors of differences in toolkit composition (site type, energy, precipitation, and raw material) to determine which individual technologies were significantly influenced by those predictors. We conducted 16 independent logistic binary regressions (one for each technology); each regression controlled for the effects of all other variables except cost path, which due its derivation was only available as a distance matrix. To control for the effects of raw material on toolkit composition, which was originally represented as a single distance matrix in our initial correlation analysis, we included the presence or absence of each raw material in each assemblage as a set of 8 independent variables (one for each raw material). Cost-path was excluded from the analysis as this variable is only avaliable as a distance matrix, which are unsuitable for logistic regression analysis.Traditional logistic regression failed to converge, due to perfect (or quasi-) separation of multiple independent variables and the relatively small size of the dataset. To ensure convergence, we employed penalized maximum likelihood using the Jeffreys invariant prior using the R package brglm [5]. All results are summarised in Supplementary Table S3.

Supplementary Table S3. Each column represents a binary logistic regression of one technology on the full set of independent variables shown as rows in the table across all 84 assemblages. The table shows positive (+) and negative (-) effects of independent variables significant at p < 0.05 (*) or p < 0.01 (**). Blanks indicate non-significant relationships. Min Sample Size is the smaller of the total number of assemblages in which the technology is present or the total number of assemblages in which the technology is absent.

|  | Backed_Microlith | BipolarTech | BladeTech | Borer | Burin | CentripetalTech | CoreTool | Denticulate | LevalloisBladeTech | LevalloisFlakeTech | LevalloisPointTech | Notch | PlatformCore | PointTech | RTBifacial | Scraper |
| --- | --- | --- | --- | --- | --- | --- | --- | --- | --- | --- | --- | --- | --- | --- | --- | --- |
| (Intercept) |  |  |  |  |  | +* |  |  |  |  |  |  |  |  |  | +* |
| Method |  |  |  |  |  |  |  |  |  |  |  |  |  |  |  |  |
| Site Type | +* | +* |  |  |  |  |  |  |  | +* |  |  |  |  |  |  |
| Simple age |  |  |  |  |  |  | +* |  |  | +* |  |  |  |  |  |  |
| Basalts |  |  |  | -** |  |  |  |  |  |  |  | -* |  |  |  |  |
| Cherts |  |  |  |  |  |  |  |  |  |  |  |  |  |  |  |  |
| CCS |  |  |  | +* |  |  |  |  |  |  |  |  |  |  |  |  |
| Obsidians |  |  | +** | +** | +* |  |  |  |  |  | +** | +* |  | +* |  | +* |
| Metamorphics |  |  | +** | +** | +* |  |  |  |  |  | +* |  |  |  | +* |  |
| Quartzs |  |  |  |  |  |  |  |  |  |  |  |  | +* |  |  |  |
| Other Igneous |  |  | +* |  |  |  |  |  |  |  |  |  |  |  |  |  |
| Other Sedimentary |  |  |  | +* |  |  |  |  |  | -** |  |  |  |  |  |  |
| Temperature |  |  |  |  |  |  |  |  |  |  |  |  |  |  |  |  |
| Precipitation | -* |  |  | -* |  | -** |  |  |  |  |  |  | -* |  |  | -* |
| Altitude |  |  |  |  |  |  |  |  |  |  |  |  | -* |  |  |  |
| Roughness | -* |  |  |  |  |  |  |  | -* | -* | -* |  |  |  |  |  |
| Min Sample Size | 32 | 35 | 26 | 32 | 14 | 29 | 37 | 15 | 10 | 17 | 19 | 33 | 40 | 27 | 15 | 21 |

S3. Palaeoreconstructions of shorelines

We used a global bathymetry model [6] with sea-level reconstructions from Spratt and Lisiecki [7] to predict the coastline for each thousand years. First, we cropped the bathymetry model to the extent of eastern Africa. To predict the palaeocoastline $h$ at any time $t$, expressed relative to the present coastline $h(t_{p})$, the equation outlined by Lambeck et al. [8] was employed:

$h\left( t \right)=h\left( t_{p} \right)-\Delta\zeta\left( t \right)$

where $\Delta\zeta\left( t \right)$ is the sea level difference at $t$ compared with the present. We mapped the coastline for each time slice onto the bathymetry model to produce a series of digital elevation models, stored in raster format in a raster stack.

S4. Downscaling approaches

To capture climatic variation across the logistical landscape of each occupation, we decided to increase the climate model beyond its native 0.5° resolution. We tested two methods of downscaling – delta downscaling [9], the bias-correction method found by Beyer *et al.* [10] to perform best at minimising the difference between empirical data and the temperature and precipitation simulations [11], and simple bilinear interpolation. Delta downscaling calculates the differences between models present and past climates and applies them to modern observed datasets to correct biases in the simulated data [10]. In this way, the delta method assumes that present day local variation remains constant through time. Using the delta-method, we downscaled the data to 2.5’ resolution (cells representing 4.625km) based on that of the modern data used, WorldClim version 2.1 (<https://www.worldclim.org/data/worldclim21.html>), and followed methods set out by Beyer et al. [10]. Alternatively, bilinear interpolation is a resampling method that disaggregates the climate model through distance weighted averaging of the four nearest cell values in order to estimate the value of a new cell. Whilst this method has been found to produce spatial artefacts [12], bilinear interpolation is commonly used in paleoclimate modelling to remap data due to its simplicity and lack of assumptions about the data. We disaggregated the model using bilinear interpolation at a factor of 12 to obtain cells representative of 4.625km resolution, matching that of the delta-downscaled data.

To test the effects of downscaling on our results, we ran the simple Mantel tests and the multiple matrix regressions on the bilinear interpolated and delta downscaled data, with sea-level estimates cropping both versions of the model according to the palaeocoastline, as well as the original climatic model at its raw resolution. As shown by Supplementary Table S4-5, the simple Mantel tests return statistically significant results for the effects of both temperature and precipitation on toolkit composition and raw material use for all versions of the climate data, except for delta-downscaled precipitation on raw material.

We then performed multiple matrix regressions to understand the independent effects of the variables on toolkit composition and raw material use (Supplementary Tables S6-7). For toolkit composition, we found that all datasets returned significant correlations between raw material, site type, roughness and precipitation (bar delta-downscaled). For raw material, toolkit composition, simple age, cost path and roughness are consistently significant, and precipitation significant for the raw data and close to significant for the bilinear interpolated data. Overall, this suggests that the delta downscaling approach is the most conservative approach, whilst using the raw data is the least conservative. In light of these results, we have opted to employ the bilinear interpolation approach to downscaling that offers increased resolution than the raw dataset but requires fewer processing steps or assumptions about how representative modern day spatial variability in local climatic variation may be for the past.

Supplementary Table S4. Simple mantel tests of the effects of precipitation on toolkit composition, raw material use and the other variables. Statistical significance highlighted at p < 0.05 (*) or p < 0.01 (**). The Benjamini-Hochberg procedure was used to adjust p values.

|  | Raw | | | Bilinear | | | Delta | | |
| --- | --- | --- | --- | --- | --- | --- | --- | --- | --- |
|  | coefficient | p | adj. p | coefficient | p | adj. p | coefficient | p | adj. p |
| Toolkit composition | 0.1956 | 0.001** | 0.003** | 0.1972 | 0.001** | 0.003 | 0.155 | 0.001** | 0.004** |
| Raw material | 0.1632 | 0.001** | 0.003** | 0.1587 | 0.001** | 0.003 | 0.0635 | 0.064 | 0.073 |
| Method | 0.0742 | 0.059 | 0.067 | 0.0957 | 0.041* | 0.047* | 0.0887 | 0.051 | 0.068 |
| Site | 0.113 | 0.002** | 0.004** | 0.0906 | 0.005** | 0.01** | 0.0835 | 0.007** | 0.013* |
| Simple age | 0.0419 | 0.163 | 0.163 | 0.0311 | 0.207 | 0.207 | -0.0342 | 0.717 | 0.717 |
| Cost path | 0.121 | 0.003** | 0.005** | 0.1127 | 0.006** | 0.001** | 0.2491 | 0.001** | 0.004** |
| Altitude | 0.2128 | 0.001** | 0.003** | 0.2071 | 0.001** | 0.003** | 0.1352 | 0.008** | 0.013* |
| Energy | 0.1138 | 0.009** | 0.012* | 0.1087 | 0.027* | 0.036* | 0.1560 | 0.004** | 0.011* |

Supplementary Table S5. Simple mantel tests of the effects of temperature on toolkit composition, raw material use and the other variables. Statistical significance highlighted at p < 0.05 (*) or p < 0.01 (**). The Benjamini-Hochberg procedure was used to adjust p values.

|  | Raw | | | Bilinear | | | Delta | | |
| --- | --- | --- | --- | --- | --- | --- | --- | --- | --- |
|  | coefficient | p | adj. p | coefficient | p | adj. p | coefficient | p | adj. p |
| Toolkit composition | 0.2365 | 0.001** | 0.001** | 0.2144 | 0.001** | 0.001** | 0.1998 | 0.001** | 0.001** |
| Raw material | 0.1452 | 0.001** | 0.001** | 0.1532 | 0.001** | 0.001** | 0.171 | 0.001** | 0.001** |
| Method | -0.0089 | 0.532 | 0.532 | 0.0077 | 0.433 | 0.433 | 0.041 | 0.219 | 0.219 |
| Site | 0.1757 | 0.001** | 0.001** | 0.1568 | 0.001** | 0.001** | 0.1376 | 0.001** | 0.001** |
| Simple age | 0.0519 | 0.12 | 0.137 | 0.0465 | 0.152 | 0.174 | 0.0734 | 0.092 | 0.105 |
| Cost path | 0.2658 | 0.001** | 0.001** | 0.2637 | 0.001** | 0.001** | 0.1932 | 0.001** | 0.001** |
| Altitude | 0.8094 | 0.001** | 0.001** | 0.8041 | 0.001** | 0.001** | 0.839 | 0.001** | 0.001** |
| Energy | 0.5037 | 0.001** | 0.001** | 0.4687 | 0.001** | 0.001** | 0.4633 | 0.001** | 0.001** |

Supplementary Table S6. Multiple matrix regression results for toolkit composition. Statistical significance highlighted at p < 0.05 (*) or p < 0.01 (**).

|  | Raw | | Bilinear interpolation | | Delta-downscaling | |
| --- | --- | --- | --- | --- | --- | --- |
|  | coefficient | p | coefficient | p-value | Coefficient | p-value |
| Raw material | 0.149 | 0.001** | 0.1483 | 0.001** | 0.155 | 0.001** |
| Method | 0.0114 | 0.629 | 0.009 | 0.698 | 0.0083 | 0.716 |
| Site type | 0.0296 | 0.016* | 0.0328 | 0.018* | 0.034 | 0.014* |
| Simple Age | 0.0157 | 0.702 | 0.0184 | 0.668 | 0.019 | 0.68 |
| Cost path | 0.0068 | 0.88 | 0.0172 | 0.669 | 0.0102 | 0.806 |
| Altitude | -0.1476 | 0.219 | -0.0896 | 0.473 | -0.0886 | 0.48 |
| Roughness | 0.2652 | 0.003** | 0.2645 | 0.004** | 0.2543 | 0.004** |
| Temperature | 0.1335 | 0.082 | 0.0672 | 0.298 | 0.0862 | 0.276 |
| Precipitation | 0.0909 | 0.031* | 0.111 | 0.02* | 0.0855 | 0.066 |

Supplementary Table S7. Multiple matrix regression results for raw material. Statistical significance highlighted at p < 0.05 (*) or p < 0.01 (**).

|  | Raw | | Bilinear interpolation | | Delta-downscaling | |
| --- | --- | --- | --- | --- | --- | --- |
|  | coefficient | p | coefficient | p | coefficient | p |
| Toolkit composition | 0.3139 | 0.001** | 0.3119 | 0.001** | 0.3242 | 0.001** |
| Method | 0.0353 | 0.1722 | 0.0351 | 0.192 | 0.0379 | 0.164 |
| Site type | -0.0056 | 0.727 | -0.006 | 0.7 | -0.0075 | 0.6426 |
| Simple Age | 0.1495 | 0.005** | 0.149 | 0.007** | 0.1404 | 0.01** |
| Cost path | 0.2055 | 0.002** | 0.1991 | 0.001** | 0.19 | 0.001** |
| Altitude | 0.2646 | 0.064 | 0.2206 | 0.106 | 0.0439 | 0.767 |
| Roughness | -0.2319 | 0.012* | -0.2286 | 0.017* | -0.2 | 0.027* |
| Temperature | -0.0575 | 0.533 | -0.0108 | 0.896 | 0.1605 | 0.091 |
| Precipitation | 0.1211 | 0.015* | 0.1124 | 0.053 | -0.0192366 | 0.737 |

**References**

[1]       Blinkhorn, J. & Grove, M. Explanations of variability in Middle Stone Age stone tool assemblage composition and raw material use in Eastern Africa. J. Archaeol. Anthropol. Sci. 13(14), (2021). [10.1007/s12520-020-01250-8](https://doi.org/10.1007/s12520-020-01250-8)

[2]       Tobler, W. Three presentations on geographical analysis and modeling. University of California, Santa Barbara, California, (1993).

[3]       van Etten, J. R package gdistance: Distances and routes on geographical grids, J. Stat. Softw. **76**(13),1-21. (2017). [10.18637/jss.v076.i13](https://www.jstatsoft.org/v076/i13)

[4]       Minetti, A. E., Moia, C., Roi, G. S., Susta, D. & Ferretti, G. Energy cost of walking and running at extreme uphill and downhill slopes, **93**(3), 1039–1046; [10.1152/japplphysiol.01177.2001](https://doi.org/10.1152/japplphysiol.01177.2001) (2002).

[5]       Kosmidis, I. ‘brglm’: Bias reduction in binary-response Generalized Linear Models. CRAN: R Packages: <https://cran.r-project.org/web/packages/brglm2/brglm2.pdf> (2007).

[6]       The General Bathymetric Chart of the Oceans (GEBCO). Gridded Bathymetry Data. Available online at: <https://www.gebco.net/data_and_products/gridded_bathymetry_data/> (2020).

[7]     Spratt, R. M. & Lisiecki, L. E. A Late Pleistocene sea level stack. Clim. Past, 12, 1079–1092, (2016). [10.5194/cp-12-1079-2016](https://doi.org/10.5194/cp-12-1079-2016)

[8]       Lambeck, K., Purcell, A., Flemming, N. C., Vita-Finzi C., Alsharekh, A. M. & Bailey, G. N. Sea level and shoreline reconstructions for the Red Sea: Isostatic and tectonic considerations and implications for hominin migration out of Africa, Quat. Sci. Rev., **30** (25–26), 3542–3574, (2011). [10.101g/j.quascirev.2011.08.008](http://dx.doi.org/10.1016%2Fj.quascirev.2011.08.008)

[9]       Maraun D. & Widmann, M. Statistical Downscaling and Bias Correction for Climate Research. Cambridge: Cambridge University Press, (2018).

[10]     Beyer, B., Krapp, M & Manica, A. An empirical evaluation of bias correction methods for palaeoclimate simulations. Clim. Past **16**, 1493–1508, (2020). [10.5194/cp-16-1493-2020](https://doi.org/10.5194/cp-16-1493-2020)

[11]     Krapp, M., Beyer, R. M., Edumundson, S. L., Valdes, P. J. & Manica, A. A statistics-based reconstruction of high-resolution global terrestrial climate for the last 800,000 years. Sci. Data **8**, 228, (2021). [10.1038/s41597-021-01009-3](https://doi.org/10.1038/s41597-021-01009-3)

[12]     Latombe, G. et al., Comparison of spatial downscaling methods of general circulation model results to study climate variability during the Last Glacial Maximum, Geosci. Model Dev. **11**, 2563–2579, (2018). [10.5194/gmd-11-2563-2018](https://doi.org/10.5194/gmd-11-2563-2018)
